# Hierarchical Information Clustering by Means of Topologically Embedded Graphs

**DOI:** 10.1371/journal.pone.0031929

**Published:** 2012-03-09

**Authors:** Won-Min Song, T. Di Matteo, Tomaso Aste

**Affiliations:** 1 Applied Mathematics, Research School of Physics and Engineering, The Australian National University, Canberra, Australia; 2 Department of Mathematics, King's College London, London, United Kingdom; 3 School of Physical Sciences, University of Kent, Kent, United Kingdom; University of Georgia, United States of America

## Abstract

We introduce a graph-theoretic approach to extract clusters and hierarchies in complex data-sets in an unsupervised and deterministic manner, without the use of any prior information. This is achieved by building topologically embedded networks containing the subset of most significant links and analyzing the network structure. For a planar embedding, this method provides both the intra-cluster hierarchy, which describes the way clusters are composed, and the inter-cluster hierarchy which describes how clusters gather together. We discuss performance, robustness and reliability of this method by first investigating several artificial data-sets, finding that it can outperform significantly other established approaches. Then we show that our method can successfully differentiate meaningful clusters and hierarchies in a variety of real data-sets. In particular, we find that the application to gene expression patterns of lymphoma samples uncovers biologically significant groups of genes which play key-roles in diagnosis, prognosis and treatment of some of the most relevant human lymphoid malignancies.

## Introduction

Filtering information out of complex datasets is becoming a central issue and a crucial bottleneck in any scientific endeavor. Indeed, the continuous increase in the capability of automatic data acquisition and storage is providing an unprecedented potential for science. However, the ready accessibility of these technologies is posing new challenges concerning the necessity to reduce data-dimensionality by filtering out the most relevant and meaningful information with the aid of automated systems. In complex datasets information is often hidden by a large degree of redundancy and grouping the data into clusters of elements with similar features is essential in order to reduce complexity [1]. However, many clustering methods require some *a priori* information and must be performed under expert supervision. The requirement of any prior information is a potential problem because often the filtering is one of the preliminary processing on the data and therefore it is performed at a stage where very little information about the system is available. Another difficulty may arise from the fact that, in some cases, the reduction of the system into a set of separated local communities may hide properties associated with the global organization. For instance, in complex systems, relevant features are typically both local and global and different levels of organization emerge at different scales in a way that is intrinsically not reducible. We are therefore facing the problem of catching simultaneously two complementary aspects: on one side there is the need to reduce the complexity and the dimensionality of the data by identifying clusters which are associated with local features; but, on the other side, there is a need of keeping the information about the emerging global organization that is responsible for cross-scale activity. It is therefore essential to detect clusters together with the different hierarchical gatherings above and below the cluster levels. In the literature there exist several methods which can be used to extract clusters and hierarchies [1]–[3] and the application to biology and gene expression data has attracted a great attention in recent years [4]–[7]. However, in these established approaches, to extract discrete clusters, one must input some a priori information about their number or define a thresholding value. This introduces other potential difficulties because complex phenomena are often associated with multi-scaling signals which cannot be trivially thresholded. In this paper, we propose an alternative method that overcomes these limitations providing both clustering subdivision and hierarchical organization without the need of any prior information, without demanding supervision and without requiring thresholding.

In recent years, several network based approaches have been proposed to describe complex data-sets and applied to several fields from biology [8], [9] to social and financial systems [10], [11]. Indeed, networks naturally reflect in their set of vertices the variety of elements in the system, they reflect in their edges the plurality of the interrelations between elements and they encode in their dynamics the complex evolution and adaptation of the system [12]–[16]. In this paper we apply the network paradigm to the study of complex data-structures. In our approach a graph with constrained complexity is built by means of a deterministic construction inserting recursively the most relevant links. In this construction, complexity is constrained by embedding the graph on an hyperbolic surface of genus 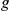 (where the genus is the number of handles of the surface) [17], [18]. The Ringel-Youngs theorem ensures that for 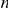 vertices the complete graph, 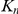, can be always embedded on a surface with large enough genus (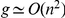) [19]. Any graph is a sub-graph of 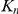 and therefore any graph can be embedded on a surface. In this paper we are interested in the limit where graphs are sparse and they are embedded on simple surfaces. The simplest case is 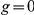 and the resulting graph is called Planar Maximally Filtered Graph (PMFG) and it is a triangulation of a topological sphere. Topologically embedded graphs on planar surfaces (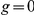) have a relatively small number of edges (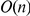) but they have high-clustering coefficients, they can display various kinds of degree distributions, from exponential to power-law tailed, and they can be used as a platform for modeling other systems [17], [20]–[23]. It has been shown that PMFG graphs are efficient filtering tools having topological properties associated to the properties of the underlying system [18], [24]. This makes the PMFG a desirable tool to extract clusters and hierarchies from complex data-sets.

## Methods

**Figure 1:**
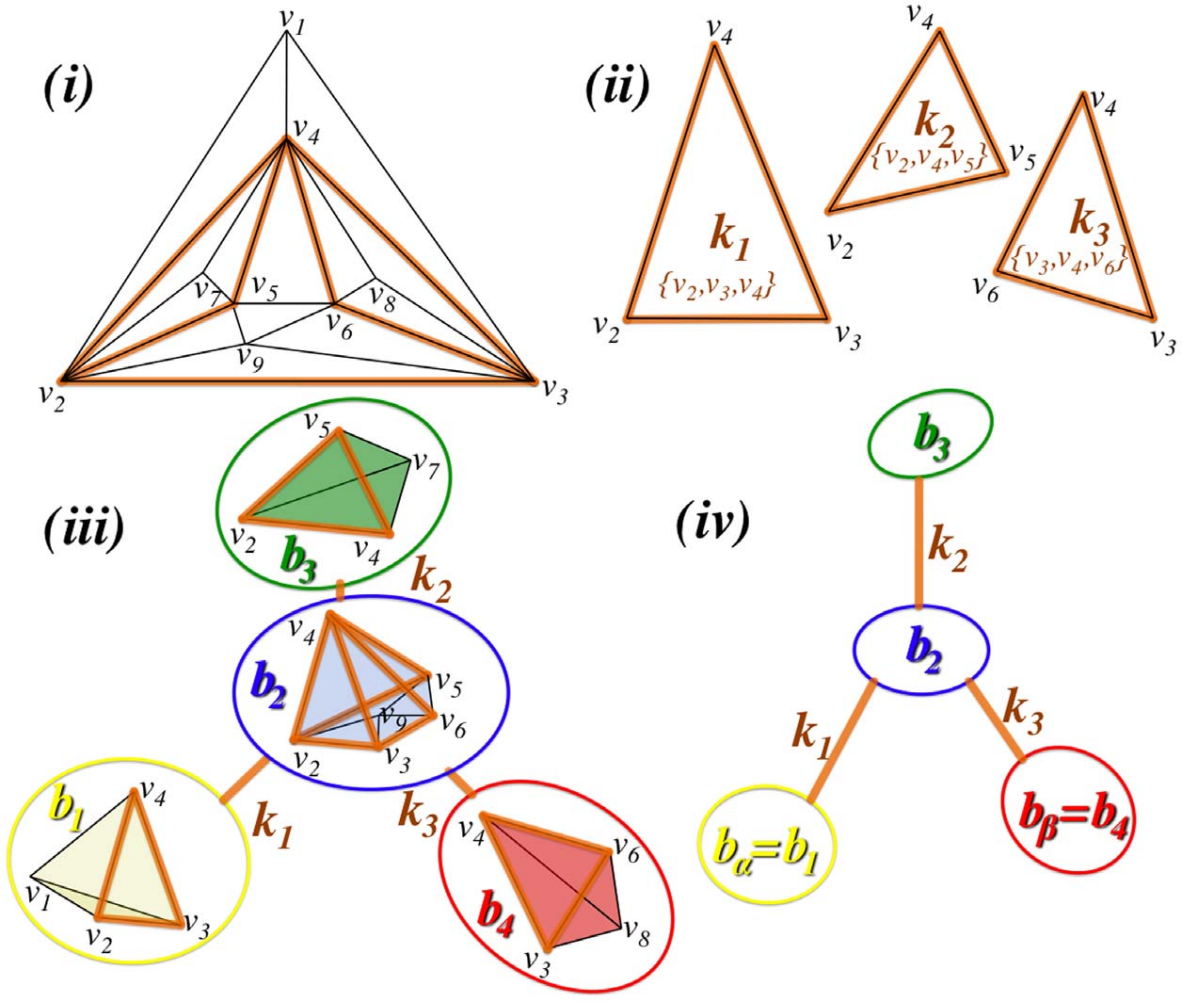
A schematic overview of the construction of the bubble tree. (i) An example of PMFG graph made of nine vertices 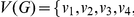 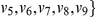 and containing three separating 3-cliques: 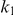, 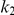 and 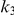. (ii) The separating 3-cliques have vertex sets: 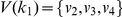, 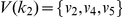, and 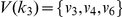. (iii) The separating 3-cliques identify four planar sub-graphs called “bubbles”: 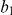, 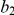, 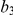 and 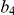 with vertex sets 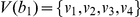, 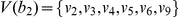, 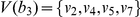 and 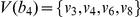. (iv) The graph can be viewed as a “bubble tree” made of four bubbles connected through three separating 3-cliques.

**Figure 2:**
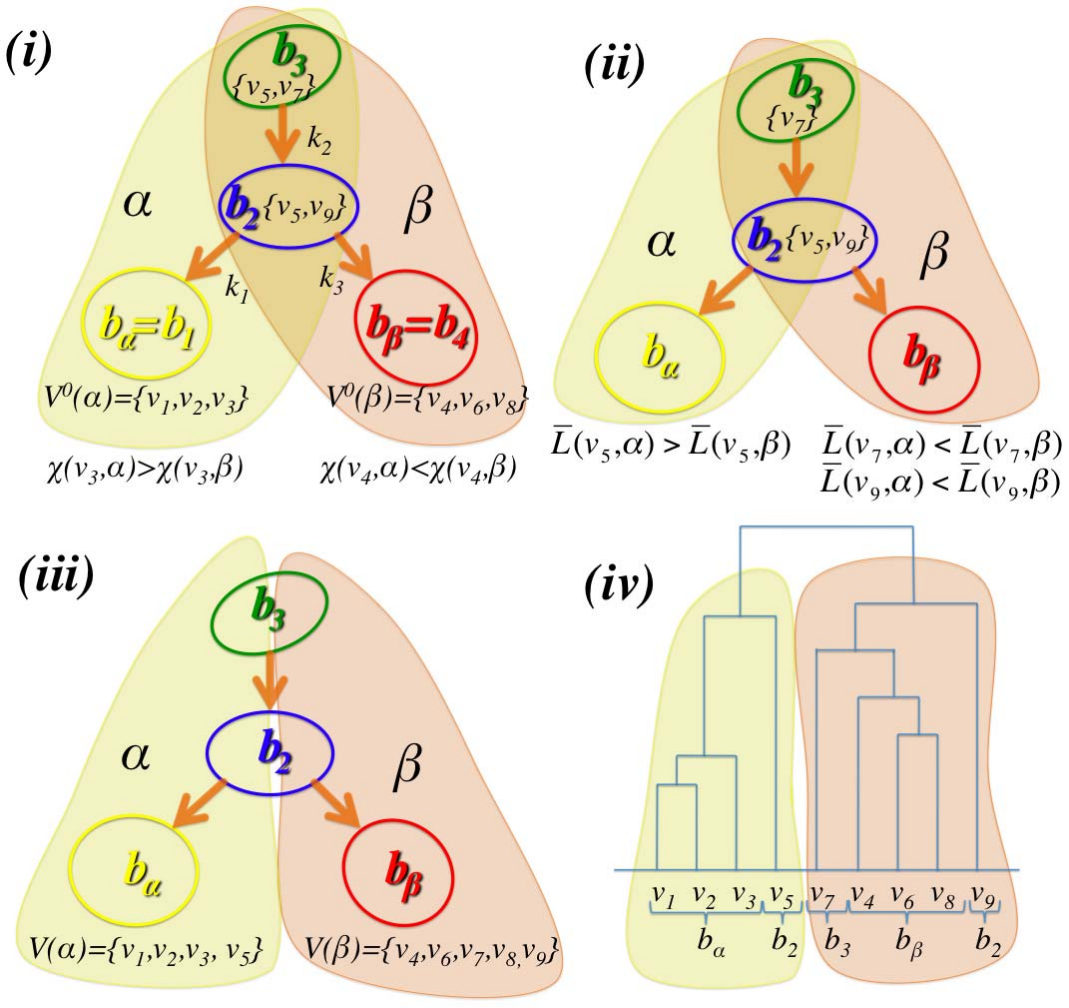
Illustration of the DBHT technique. (i) Construction of the directed bubble tree where directions are given to the 3-cliques 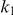, 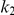 and 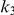 (from Fig. 1) accordingly with the largest weight 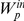 and 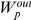 (see Eq.1). In this example we have two converging bubbles: 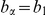 and 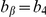. A unique set of vertices can be associated to each of the two converging bubbles 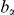 and 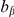 where vertices shared by both the converging bubbles (i.e. the vertices 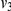 and 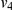) are assigned accordingly with the largest strength 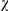 (Eq.2). (ii) All the other non-assigned vertices (i.e. 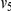, 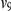 and 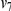) are associated to the cluster with minimum average shortest path length 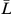 (Eq.3). (iii) The vertex set is uniquely divided into two clusters respectively associated to the two converging bubbles: 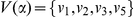 and 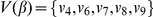. (iv) The hierarchical organization and the clustering structure can be represented with a dendrogram.

The general idea at the basis of our method is to use the topological structure of PMFG graphs to investigate the properties of the data-sets. The PMFG is a weighted graph where edges 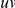 have weights 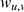 which, in general, are similarity measures (a larger weight 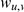 of edge 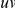 corresponds to a stronger similarity between 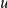 and 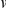). Furthermore, a distance 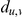, or more generally, a non-negative dissimilarity measure is also associated to the edges. Specifically, the PMFG is a graph 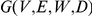 where 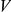 is the vertex set, 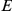 the edge set, 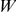 the edge-weight set and 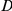 the edge-distance set. A hierarchy in 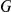 can be built from a simple consequence of planarity which imposes that any cycle (a closed simple path with the same starting and ending vertex) must be either separating or non-separating [25]. If we detach from the graph the vertices belonging to a separating cycle then two disjoint and non-empty subgraphs are produced. The simplest cycle is the 3-clique which is a key structural element in PMFGs. An example of PMFG is shown in Fig. 1 where the separating 3-cliques are highlighted. By definition, each separating 3-clique, 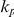, divides the graph 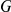 into two disconnected parts, the *interior* 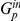 and the *exterior* 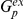, that are joined by the clique itself. The union of one of these two parts and the separating clique is also a maximally planar graph. Such a presence of cliques within cliques provides naturally a hierarchy. The subdivision process can be carried on until all separating 3-cliques in 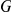 have been considered. The result is a set of planar graphs, that we call “bubbles”, which are connected to each other via separating 3-cliques, forming a tree [26]. In Fig. 1(iv) the “bubble tree”, denoted hereafter 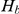, and its construction are shown. In the bubble tree vertices 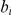 represent bubbles and edges 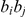 represent the separating 3-clique, 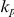, which is connecting the two bubbles. A direction can be associated to each edge in 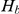 by comparing the sums over the weights of the edges in the PMFG connecting the 3-clique 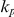 with the two bubbles. Specifically, a direction can be associated to the edge 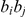 by comparing the connections of 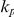 with the interior sub-graph 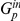 and the exterior sub-graph 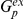 and considering the two weights
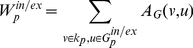
(1)where 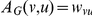 is the adjacency matrix of 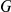. The direction is given toward the side with largest weight obtaining 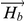. (In the case of equal weights in the two directions, the two bubbles are joined into a single larger bubble.) In 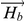 there are three different kinds of bubbles: (1) *converging bubbles* where the connected edges are all incoming to the bubble; (2) *diverging bubbles* where the connected edges are all outgoing from the bubble; (3) *passage bubbles* where there are both inwards and outwards connected edges. An example is provided in Fig. 2 where we have two converging bubbles (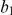 and 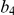), one diverging bubble (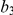) and one passage bubble (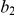). Converging bubbles are special being the end points of a directional path that follows the strongest connections and we consider them as the centers of clusters. Any bubble 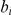 connected by a directed path in 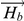 to a converging bubble 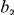 belongs to cluster 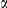. By construction, bubbles in cluster 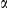 form a subtree 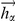 which has only one converging bubble 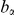 and all edges are directed toward 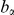. This is a non-discrete clustering of bubbles because there can be multiple directed paths between 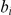 and two or more converging bubbles 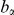, 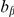,… . In Fig. 2(ii) the two subtrees converging toward 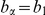 and 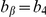 are highlighted, it is clear that in this example bubbles 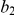 and 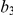 are shared by the two subtrees. A non-discrete clustering of the vertex set 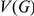 can now be obtained by assigning to each vertex 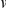 the cluster memberships of the bubbles that contain it. In order to obtain a *discrete* clustering for 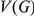, we uniquely assign each vertex to the converging bubble which is at the smallest shortest path distance (see Fig. 2 for a schematic overview). This is achieved in two steps. *First*, we consider the vertices in the converging bubbles. Some vertices belong to only one converging bubble and, in this case, they are assigned to it (e.g. in Fig. 2 vertices 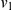 and 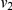 are assigned to 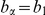 and vertices 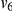, 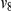 are assigned to 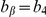). Other vertices instead belong to more than one converging bubble (e.g. vertices 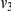 and 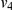 in Fig. 2) and in this case we look at the ‘strength’ of attachment
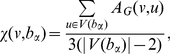
(2)and assign each vertex to the bubble with largest strength. (The notation 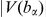 in Eq.2 indicates the number of vertices in the vertex set of 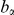 and 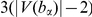 is the number of edges in the bubble.) After this assignment, each converging bubble 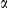 has a unique set of vertices 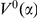. (There can be converging bubbles with an empty set of vertices and, in this case, there will be no clusters associated to them.) *Second*, we consider all the other remaining vertices (e.g. vertices 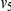, 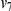 and 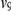 in Fig. 2). A vertex 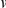 may belong to more than one subtree 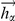, 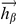
… and, in this case, it is assigned to the converging bubble that has the minimum mean average shortest path distance
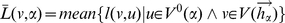
(3)with respect to all other converging bubbles. Here 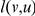 is the shortest path distance on 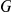 from 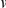 to 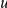 (the smallest sum of distances 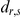 over any path between 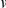 and 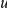). We have now obtained a discrete partition of the vertex set 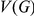 into a number of sub-sets 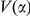, 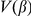,… each respectively associated to the converging bubbles 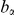, 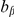,… .

Once a unique partition of the vertex set into discrete clusters has been obtained, we can investigate how each of these clusters is internally structured and how different clusters gather together into larger aggregate structures. This can be achieved with a specifically tailored linkage procedure that builds the hierarchy at three levels.

1. *Intra-bubble hierarchy:* we first assign each vertex 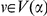 to a bubble 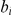 in the subtree 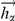. Vertices in the converging bubbles have been already assigned to the sets 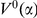. For all remaining vertices, the ones belonging to only one bubble are assigned to such bubble (e.g. vertices 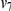 and 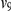 in Fig. 2). Whereas, vertices that belong to more than one bubble (e.g. vertex 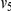 in Fig. 2) are assigned to the bubble that maximizes the strength 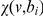 (Eq.2). In this way for every cluster 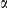 and for each bubble 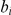 in 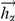 we have a unique vertex set 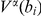 on which we can now perform a complete linkage procedure [27] by using the shortest path distances 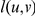 as distance matrix.
2. *Intra-cluster hierarchy:* we perform a complete linkage procedure between the bubbles in 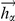 by using the distance matrix
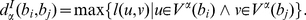
(4)
3. *Inter-cluster hierarchy:* we perform a complete linkage procedure between the clusters by using the distance matrix
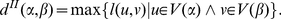
(5)

With this procedure we obtain a novel linkage that starts from the discrete clusters and at higher level joins the clusters into super-clusters and, instead, at lower level splits the clusters into a hierarchy of bubbles and splits the bubbles into a hierarchy of elements. For brevity, in the rest of the paper, we will refer to our clustering and linkage method as the *DBHT technique*.

The computational complexity of this method is smaller than 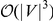 (with 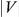 the number of vertices, which is equal to the number of variables in the dataset) and it is dominated by the construction of PMFG. Indeed, the Boyer-Myvold algorithm to check planarity [28] runs in 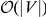 and it might have to be run for each couple of vertices (i.e. 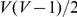 times). However, typically, the algorithm terminates before the exhaustive scanning of all edges. From empirical tests, performed on various datasets, we measured an overall runtime of 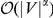 with 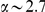. (See Supporting Information S1 and S2.)

## Results

In this section, we apply the DBHT technique to various data sets ranging from artificial data with known clustering and hierarchical structures to real gene expression data. Comparisons are made between the results retrieved by the DBHT technique and some of state-of-the-art cluster analysis techniques such as k-means++[29], Spectral clustering via Normalized cut on k-nearest neighbor graph (kNN-Spectral) [30], [31], Self Organizing Map (SOM) [32] and Q-cut [33]. Let us here stress that all these techniques –except DBHT– are non-deterministic and require some *a priori* information in order to setup the initial parameters. To compare with the DBHT technique, we run the other techniques for a broad range of parameters and pick the set of parameters that are best performing in average. This is an important negative bias against the DBHT technique that however, as we shall see shortly, can still outperform consistently the state-of-the-art counterparts. We also tested the capability of DBHT technique to correctly detect the hierarchical organization by applying it to known synthetic datasets and comparing the results with the outcomes from average and complete linkage techniques. Furthermore, we explored the meaningfulness of the hierarchical gathering of clusters and the significance of their subdivision in sub-clusters by looking at the functional properties of these gatherings and splittings in real datasets.

### Tests DBHT clustering on synthetic data

We have evaluated performance of the clustering techniques by comparing their outcomes with the known artificial clustering structure by using a popular external validity index: the adjusted Rand index [34] which returns 1 for a perfect match and in average 0 for a random guess. Specifically, we have generated correlated data-series by using a multivariate Gaussian generator (MVG) [35] that produces 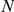 stochastic time series 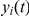 of length 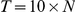 with zero mean and Pearson's cross-correlation matrix 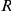 that approximates an input correlation structure 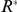 which is a block-diagonal matrix where the blocks represent the clusters and may have different sizes. The matrix 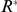 has all ones on the diagonal, it has zero correlations outside the blocks (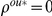) and it has a correlation value 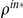 inside the blocks. Furthermore, we have added a number 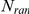 of random correlations unrelated to the cluster structure. We have also generated multivariate Log-Normal distributions by taking the exponential of MVG series generated by using reference correlation 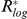 which is devised to retrieve the correct approximation of 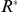 with log-normal statistics [36]. To these correlated series we have added a noise 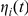 obtaining 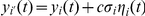, where 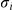 is the standard deviation of 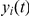 and 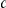 is a constant that can be used to tune the relative amplitude of noise. We have tested normally distributed (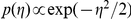), log-normally distributed (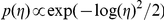) or power-law distributed (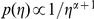) noises. We have used different values for the relative amplitude of noise 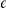 and, in the case of power-law distributed noise, we have also varied the exponent 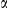. By increasing the effect of noise and/or the number of random elements, the Pearson's cross-correlation matrix 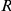 passes from a very well defined structure similar to 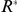 to a less defined structure where the difference between the average measured intra- and inter-cluster correlations in 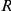, 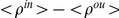, becomes negligible.

**Figure 3:**
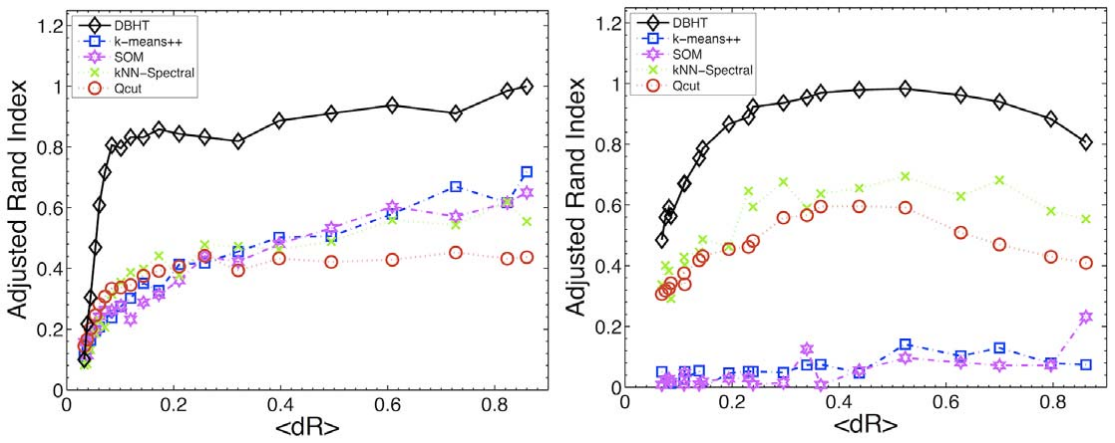
Demonstration that the DBHT technique can outperform other state-of-the-art clustering techniques, namely: k-means++[29], Spectral clustering via Normalized cut on k-nearest neighbor graph (kNN-Spectral) [30], [31], Self Organizing Map (SOM) [32], and Q-cut [33]. The figures report the adjusted Rand indexes [34] for the comparison between the the ‘true’ partition embedded in the artificially generated data and the partition retrieved by the clustering methods. In these examples we have eight clusters of size 5 elements and one cluster of size 64 elements with 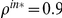, 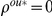 and 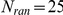. The plots report average values over a set of the 30 trials. The horizontal-axis reports the gap between average intra- and inter-cluster correlations 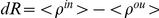 that becomes smaller when the noise 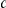 increases. (**a**) Normally distributed correlated datasets with added Normal noise with 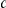 varying from 0 to 4. (**b**) Log-Normally distributed correlated datasets with added power law noise with 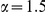 and 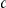 varying from 0 to 0.1.

Figure 3 compares the performance of the DBHT technique with k-means++, SOM, kNN-Spectral and Q-cut for correlated synthetic datasets consisting of 129 data series generated both with normal and log-normal statistics, with normal or power law noise with 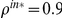, 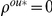 and 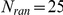. This example refers to a rather extreme case where the clusters have highly dis-homogeneous sizes with one large cluster with 64 elements and eight clusters with 5 elements each. As one can see from Fig. 3 in this case the DBHT technique is strongly outperforming the other methods. In the Supporting Information S1, we report on a large number of cases where we demonstrate that consistently the DBHT technique is better, or at least equivalent, to the best performing counterparts for a very broad range of combinations of different kinds of artificial data. Let us here note that stochastic techniques such as k-means++ and SOM are particularly sensitive to noise distributions and tend to perform poorly with fat-tailed distributed noise. On the other hand, the Qcut technique carries an inherent resolution limit that over-shadows small clusters [37]. The DBHT technique instead is less affected by these factors and it consistently delivers good performances across the range of parameters.

### Tests DBHT hierarchy on synthetic data

**Figure 4:**
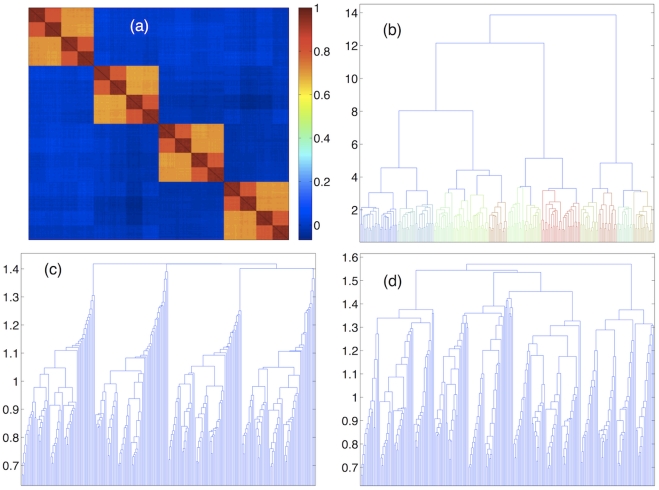
Demonstration that the DBHT technique can detect clusters at different hierarchical levels outperforming other established linkage methods. The synthetic data are generated via a multivariate Gaussian generator with added power law noise with exponent 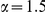 and 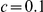. (**a**) Input correlation 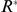 for a synthetic data structure with nested hierarchical clustering with 4 ‘large’ clusters, containing 8 ‘medium’ clusters, containing 16 ‘small’ clusters. (**b**) Dendrogram associated with the DBHT hierarchical structure. (**c**) Dendrogram associated with the Average linkage. (**d**) Dendrogram associated with the Complete linkage.

We have tested the capability of the DBHT technique to detect hierarchies by simulating data with hierarchical structure such that smaller clusters are embedded inside larger clusters making a nested structure with different intra-cluster correlations. An example is shown in Fig. 4(a) where we report an input correlation 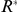 which is a nested block-diagonal matrix with zero inter-cluster correlation and with a structure of 4 ‘large’ clusters (64 elements each) with intra-cluster correlation of 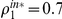. Each of the large clusters contains inside two ‘medium’ clusters (8 in total with 32 elements each) with 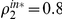 that contain inside two ‘small’ clusters (16 in total with 16 elements each) with 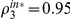. We have simulated 30 different sets of data series of length 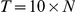 by using MVG from 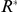 with added power law noise with 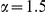 and 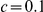. We have tested the efficiency of the DBHT technique by moving through the hierarchical levels varying the number of clusters from only one at the top hierarchy to the number of elements at the lowest hierarchy. Fig. 4(b) shows the dendrogram retrieved with the DBHT technique. By following the hierarchy from top to bottom, one can see that a structure with 4 main clusters rapidly emerges and its partition coincides exactly with the ‘true’ partition in 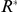. Then these clusters correctly split into two parts each making 8 clusters in total scoring a value of 0.97 for the adjusted Rand index with respect to the ‘true’ partition at this level. Finally, these 8 clusters split again producing a partition that has an adjusted Rand index of 0.94 with respect to the ‘true’ partition at this level. The partition into discrete clusters identified by the DBHT is almost identical with this last one having 17 clusters instead of the 16 ‘true’ clusters and achieving also an adjusted Rand index of 0.94 (see Supporting Information S1). One can see from Fig. 4(c,d) that, instead, the complete and average linkages give a less clear hierarchical structure. Several other examples are reported in the Supporting Information S1. The better performance of the DBHT technique over linkage methods can be explained by the fact that linkage techniques suffer from the greedy nature of the algorithm, where a misclassification of an element in an early stage of clustering can never be remedied [1], [3]. The rate of misclassification depends on the type of linkage distance, with the average linkage optimized for isotropic clusters, and complete linkage optimized for compact and well-defined clusters. On the other hand, DBHT hierarchy is based on a combination of linkage distance and topological constraints at multiple hierarchical levels: bubbles, clusters, bubble tree. This reduces the error rate with respect to the complete linkage distance.

### Application of DBHT technique to Fisher's Iris Data

**Figure 5:**
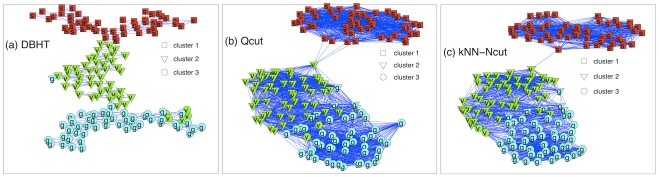
Comparison between the clustering obtained via: (a) DBHT technique, (b) best Qcut and (c) best kNN-Spectral on iris flower data set from Fisher [38]. The labels inside the symbols correspond to the three different types of flowers: (s) Iris Setosa; (v) Iris Versicolour; (g) Iris Virginica. The shapes of the symbols correspond to the clusters retrieved by the different clustering techniques.

One of the typical benchmark referred in clustering analysis literature is the iris flower data set from Fisher [38]. Briefly, the data set contains the measure of four features (i) sepal length; (ii) sepal width; (iii) petal length; (iv) petal width, for 50 iris plants from three different types of iris, namely (1) Iris Setosa; (2) Iris Versicolour; (3) Iris Virginica. The data set is available from UCI Machine Learning Repository website [39]. It is known that, the clustering structure of the data set linearly separates one type of Iris from the other two. The remaining two types are instead not linearly separable and their subdivision is a classical challenge for any clustering technique [39]. Here, in order to compute clustering and hierarchies we have used the pair-wise Euclidean distance 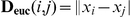 as dissimilarity matrix and 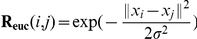 as similarity matrix [31], where 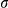 is the standard deviation of 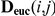 for all pairs of 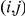. From these measures, we directly computed clustering and hierarchies via DBHT technique obtaining the graph structure shown in Fig. 5(a) where one can see that all the three iris types are rather well separated occupying different parts of the graph. By extracting three clusters from the DBHT hierarchy we observe that the first flower type (Iris Setosa) is fully separated and the other two are rather well divided with only a few misplacements. The DBHT results are compared with other two graph-based techniques, Qcut and kNN-Spectral techniques computed using 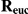 for a range of 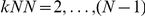. These methods are non deterministic and we retained only the best partitions which give the highest adjusted Rand score which are shown in Fig. 5(b,c). We can observe that Qcut and kNN-Spectral techniques provide a poorer separation of the last two flower types (Iris Versicolour and Iris Virginica). This is quantified by the adjusted Rand index computed by comparing with the true partition that gives 0.89 for DBHT and 0.85 for both Qcut and kNN-Spectral. Indeed, these last two techniques both misplace 8 elements of the two groups whereas DBHT misplaces only six. Other two clustering techniques, k-means++ and SOM, have been run over 30 iterations with an input number of clusters 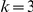, yielding to poorer partitions with the largest adjusted Rand indexes respectively of 0.73 and 0.80 which are well below the score achieved by the DBHT technique. The iris flower data set and the codes to reproduce the result in Fig. 5(a) are provided in the Supporting Information S2.

### Application of DBHT technique to a benchmark gene expression dataset

In order to validate the applicability of DBHT technique on gene expression data, we have used a benchmark dataset collected by de Souto *et al* [40] which contains 21 Affymetrix and 14 cDNA gene expression patterns from different cancer types. For this dataset we have compared clusters computed via DBHT technique with clusters computed with k-means++, kNN-Spectral, and Qcut evaluating the respective performances by using the Adjusted Rand index [34]. Differently from DBHT that requires no prior parameters, k-means++ and kNN-Spectral require instead information on the number of clusters, and we therefore tested two cases: (i) clustering with benchmark number of clusters given *a priori*; (ii) clustering with an internal validity measure to estimate the optimal number of clusters (namely: Dunn index [41], Davies-Bouldin [42] and Silhouette width [43], see Ref. [43]). Another requirement for kNN-Spectral is the number of nearest neighbors 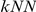. We have used 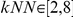 (as indicated in Ref. [31]) and picked the case with best mean performance. Also Qcut requires to choose the value of 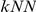. In this case, we have used 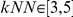 (as suggested by Ref. [33]) and selected the value which yields to the best 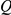.

**Figure 6:**
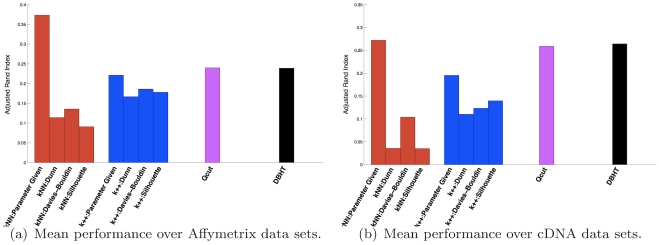
Average Adjusted Rand index to compare performances of clustering algorithms: k-means++, Qcut, kNN-Spectral and DBHT for the benchmark data sets collected by de Souto *et al* [40] (k++ indicates k-means++). The relatively high performing “Parameter given” results refer to cases when the true number of cluster is given to the algorithm as input. In all the other cases the number of cluster is computed by using internal validity measures. (a) Affymetrix data; (b) cDNA data.

**Figure 7:**
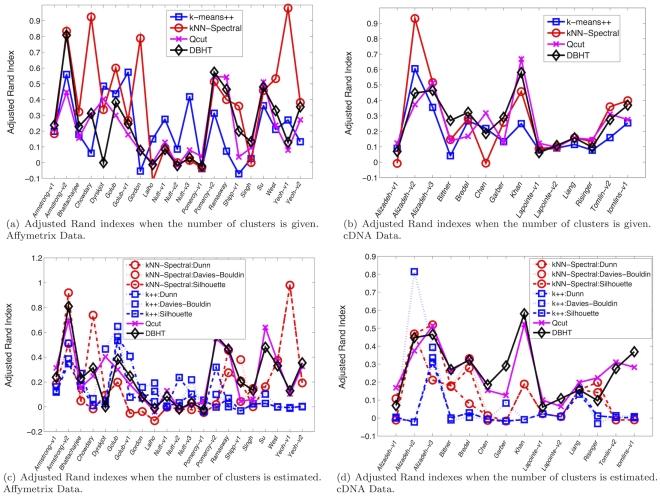
Adjusted Rand indexes for each sample in the de Souto *et al* [40] datasets. (Top) Performances for each dataset when the true number of cluster is given as input. (Bottom) Performances for each dataset when the true number of cluster is computed by using internal validity measures. (Left) Affymetrix data; (Right) cDNA data.

The results are shown in Fig. 6. We can see that DBHT and Qcut achieve the best average performances when the number of clusters is not given as input. Instead, when the benchmark number of clusters is supplied, then kNN-Spectral shows the best mean performances, followed by DBHT and Qcut that perform similarly, and finally k-means++. Let us stress that the true number of clusters is an important piece of information that is not available in most practical cases and therefore an high performance in this case may not be of practical relevance. However, we note that, even in this unfavorable case, the DBHT can perform extremely well. Indeed, if we look at the performances for each sample (see Fig. 7) we see that DBHT can achieve the best performance for many cDNA data and for some Affymetrix data.

**Figure 8:**
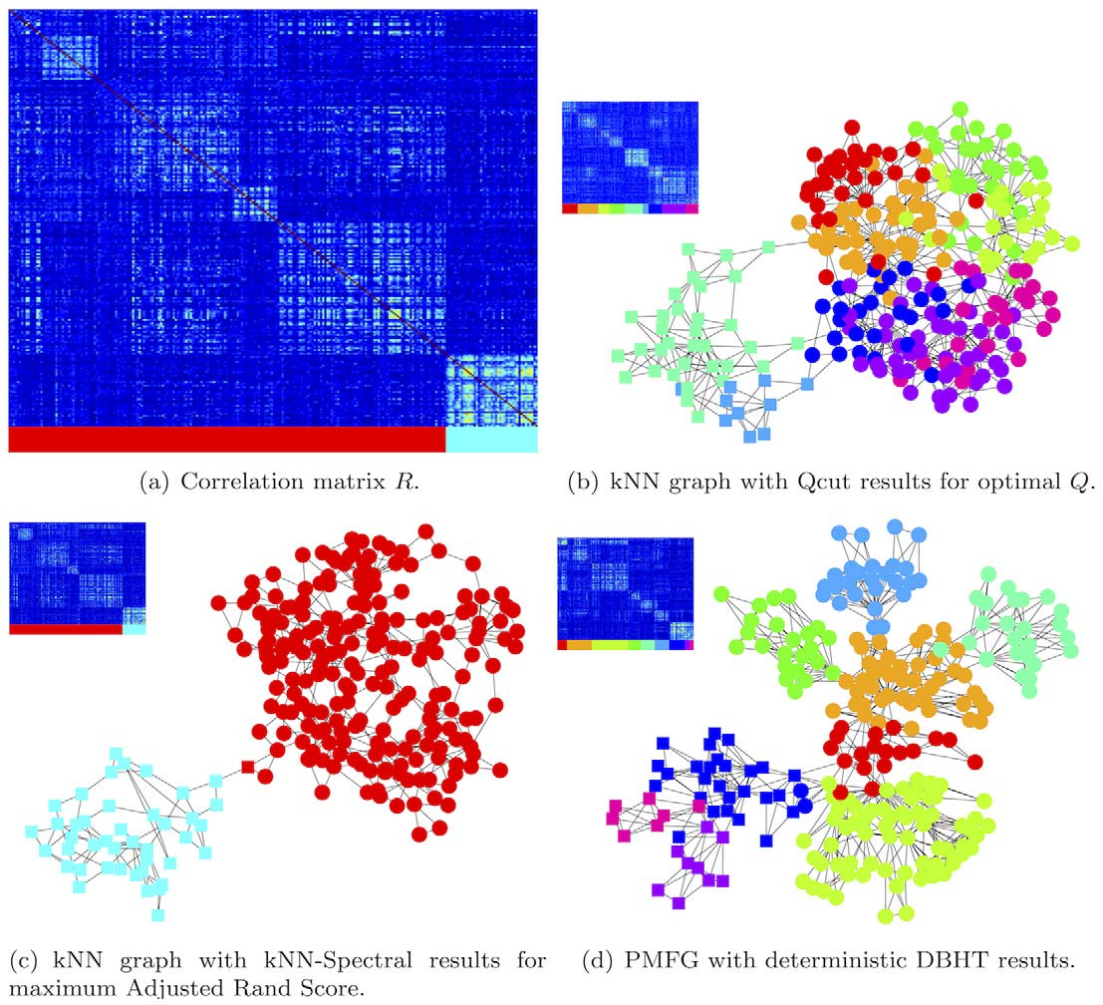
Comparison between the clusters obtained with the DBHT method and the clusters obtained from kNN graph with Qcut results for optimal Q for the dataset Yeoh-v1 Affymetrix [40]. (a) Correlation matrix structure 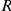, which are ordered accordingly with the ‘known’ clustering structure of Yeoh-v1 data. (b, c, d) *Insets*: correlation matrices 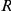 ordered accordingly with the Qcut, kNN-Spectral and DBHT respectively. The clusters are indicated on the bottom with color bars. (b, c, d) *Main plots*: results for Qcut, kNN-Spectral and DBHT respectively where the ‘golden standard’ clusters for Yeoh-v1 data (as by de Souto *et al* [40]) are depicted in vertices of different shapes: square or circle. The computed clusters are instead depicted in different colors, shown both in the graphs and in the color bars on the bottom of the Correlation matrix. One can note that, despite kNN-Spectral technique gives a very good agreement with the ‘golden standard’ provided by de Souto *et al*, the structure extracted by the DBHT method gives a very clean clustering partition that is clearly revealed in the visualization of the relative correlation matrix in the inset of (d).

Let us remark that, the ‘golden standard’ clusters provided by de Souto *et al* do not necessarily represent the true and meaningful underlying structure of the gene expression data. For example, in Fig. 8, we have analyzed in details the special case of Yeoh-v1 Affymetrix data which gives outstanding performance for kNN-Spectral technique and poorer performance for the DBHT technique (see Fig. 7). Fig. 8(a) shows the correlation structure 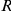 of the data set visualized according to the known golden standard cluster structure. One can see that, beside the golden standard clusters, there is also a finer meaningful structure that is not detected by kNN-Spectral but it affects instead the DBHT clustering. In this case, the high performance of the kNN-Spectral is a consequence of a coarse-grained picture which is not necessarily best reflecting all the features of the dataset. In general, in practical cases, the real clustering structure is often ambiguous and the availability of clustering methods based on different criteria is a key ingredient to properly explore these structures. Often, the subdivision into distinct clusters is not well defined and the information provided by the DBHT thechnique, concerning the hierarchical way in which clusters split into sub-parts and in which they merge into larger aggregates, can become essential.

### Application of DBHT technique to gene expression data set from human cancer samples

We have applied the DBHT technique to analyze gene expression data sets collected by Alizadeh *et al* [44] concerning 96 malignant and normal lymphocyte samples belonging to the three most relevant adult lymphoid malignancies, namely: Diffuse Large B-Cell Lymphoma (DLBCL); Follicular Lymphoma (FL); Chronic Lymphocytic leukemia (CLL); together with other 13 kinds of samples from normal human tonsil, lymph node, Transformed Cell Line, Germinal Centre B, Activated Blood B, and Resting Blood B. This data set has already served as a benchmark to evaluate performance of clustering techniques on gene expression data [33], [45] and this is why we have chosen to test our method on this referential dataset. Patients with DLBCL cancer type have variable clinical courses and different survival rates and there are strong indications that DLBCL classification includes more than one disease entity [44]. The challenge for a clustering algorithm is therefore to analyze the DLBCL genetic profiles and individuate different subtypes of DLBCL to be associated with different clinical courses. Indeed, various studies have attempted to highlight genetically significant genes that can be of clinical significance to improve the DLBCL patients' diagnosis and clinical treatments [44], [46]–[51]. In particular, it is understood that DLBCL is a very heterogeneous type of Lymphoma and there are at least three distinct subtypes which differ in treatment methods for improved survival of the patients [44], [46], [52].

**Figure 9:**
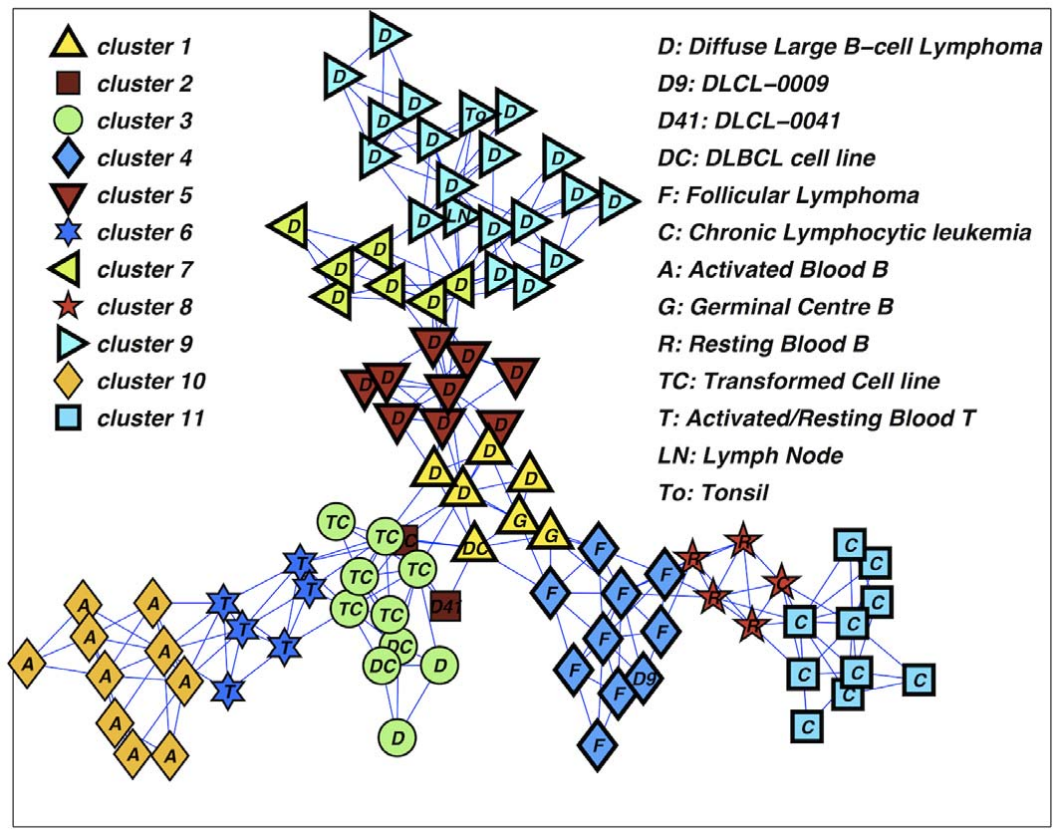
Sample-cluster structure for 96 malignant and normal lymphocyte samples from Alizadeh *et al* 2000 [44], the labels inside the symbols correspond to the different sample types as listed in the legend. The DBHT technique retrieves 11 sample-clusters here represented with different symbols (see legend). The underlying network is the PMFG from which the clustering has been computed.

We have first applied the DBHT technique on the gene expression data by using Pearson's correlation as similarity measure, and correlation distance as the dissimilarity measure. The DBHT clustering yielded to 11 sample-clusters, which are shown in Fig. 9. One can immediately note that all FL samples are gathered together in one cluster that also contains the DLCL-0009 sample, which has been associated to FL in other studies on the same data [33], [44]. Transformation of FL to DLBCL is common [53], and this cluster suggests that DLCL-0009 may have derived from FL, sharing therefore common gene expression patterns. We also observe in Fig. 9 that all, except one, the CLL samples occupy a single cluster. The missing CLL sample is attached to this cluster and it is included in a cluster containing Resting Blood B samples which have indeed similar expressions patterns and clinical similarity to CLL and are often merged together by other clustering techniques [33]. DLBCL cancer types appear in four different sample-clusters which are however lying together in a branch of the PMFG graph. Significantly, these clusters also include some other GCB-like samples. Remarkably, if we look at the patient survival rates (Table 1), we see that these four sample-clusters are extracting DLBCL cancer subtypes with very different clinical courses. Indeed, if we consider separately the patients with DLBCL type of Lymphoma accordingly with the subdivision into the four sample-clusters ‘1’, ‘5’, ‘7’ and ‘9’ (from bottom to top of the Fig. 9), they respectively have survival rates 100%, 56%, 15% and 29% (see Table 1 for details). In the work of Alizadeh *et al* [44] survival rate differentiation in DLBCL patients was associated with two main cancer subtypes, namely GCB-like and ABC-like, with the latter considered more fatal than the former. We can note that, in our clustering, sample-cluster ‘1’ contains GCB-like DLBCL, and it also includes other GCB samples such as tonsil GCB, tonsil GC fibroblast, and high survival rates are common in GCB-like cancer types (see Supporting Information S1). Cluster ‘5’ is also characterized by GCB-like DLBCL samples, however its proximity to ABC-like clusters (see Supporting Information S1), may be the clue to relatively low survival rate in comparison to cluster ‘1’. Cluster ‘9’ is characterized by a majority of ABC-like DLBCL to which we may attribute its relatively low survival rate [44]. On the other hand, cluster ‘7’, which shows a surprisingly low survival rate, has instead a significant number of GCB-like DLBCL samples, this might signal the existence of another relevant DLBCL subtype. The gene expression data and the codes to reproduce the result in Fig. 6 are provided in the Supporting Information S2.

**Table 1:** Survival rates of cancer patients with DLBCL type of Lymphoma. The patients are divided in four groups corresponding to the four sample-clusters containing DLBCL obtained with DBHT technique (see Fig. 6).

	Sample Cluster ‘1’	Sample Cluster ‘5’	Sample Cluster ‘7’	Sample Cluster ‘9’
Cluster Size	7	9	7	20
# of DLBCL	4	9	7	17
# Survived over 5 yrs	3 (100%)	5 (56%)	1 (14%)	5 (29%)
# Died in 5 yrs	0	4	6	12

In order to functionally validate these sample-clusters, we have analyzed the expression profiles for 6 groups of genetic clones with known physiological roles, namely: GCB- Germinal Center B cell (111 clones), LyN- Lymph Node (136 clones), PBC- Pan B Cell (81 clones), Pr- Proliferation (312 clones), TC- T Cell (111 clones) and ABC- Activated B Cell (86 clones) [44]. The significance of regulation patterns has been evaluated by one-tailed T tests with cut-off p-value of 0.01. The number of up-/down-regulated profiles for each group of clones is shown in Table 2. Significant up-/down-regulation patterns of the expression profiles in the sample-clusters reflect the biological relevance the group of gene-clones in each sample-cluster. We first observe that sample-clusters containing DLBCL cancer types (e.g. cluster ‘1’, ‘5’, ‘7’ and ‘9’) distinguish from other samples by up-regulating more clones from Pr, hence reflecting higher proliferative index. Sample clusters associated to DLBCL are also differentiating among themselves, for instance, sample-clusters ‘1’ and ‘5’ both up-regulate GCB clones but they differ significantly in the up-regulation of LyN clones, supporting the subdivision of GCB-like DLBCL by these sample clusters. Similarly, sample-cluster ‘7’ shows a unique expression signature that highlights a strong up-regulation of LyN clones in comparison to other clones. Given that this sample-cluster is a mixture of ABC-like and GCB-like DLBCLs, and it shows distinctively low survival rate, this again suggests that sample-cluster ‘7’ is a different subtype of DLBCL outside of GCB-/ABC-like classification. Overall, these results indicate that DBHT clustering technique is able to reveal a meaningful classification of biologically significant DLBCL subtypes which is richer than what proposed in the original study by Alizadeh *et al* [44].

**Table 2:** Number of up-regulated (on the left) and/down-regulated (on the right) expression profiles for each group of clones with known physiological roles as reported in Ref. [44].

	GCB	LyN	PBC	Pr	TC	ABC
Sample Cluster ‘1’	**61/0**	0/2	27/0	**115/0**	1/15	4/12
Sample Cluster ‘2’	2/0	0/2	0/2	7/3	0/1	0/3
Sample Cluster ‘3’	0/35	2/37	0/15	259/0	0/38	4/3
Sample Cluster ‘4’	83/0	0/97	48/0	1/193	3/12	0/37
Sample Cluster ‘5’	**21/2**	97/0	7/3	**119/0**	2/4	0/11
Sample Cluster ‘6’	7/27	1/47	0/61	6/126	86/0	32/0
Sample Cluster ‘7’	4/6	**111/0**	0/24	1**7/4**	14/3	13/1
Sample Cluster ‘8’	0/2	0/41	17/1	0/199	6/4	2/7
Sample Cluster ‘9’	1/13	133/0	7/1	**70/0**	14/4	24/2
Sample Cluster ‘10’	0/37	3/48	1/14	44/68	1/20	61/0
Sample Cluster ‘11’	20/43	0/110	27/12	0/303	20/16	1/56

The sample-clusters are obtained by DBHT technique and labels are as in Fig. 9. Some significant up-/down-regulation patterns, commented in the text, are highlighted by boldface font.

**Figure 10:**
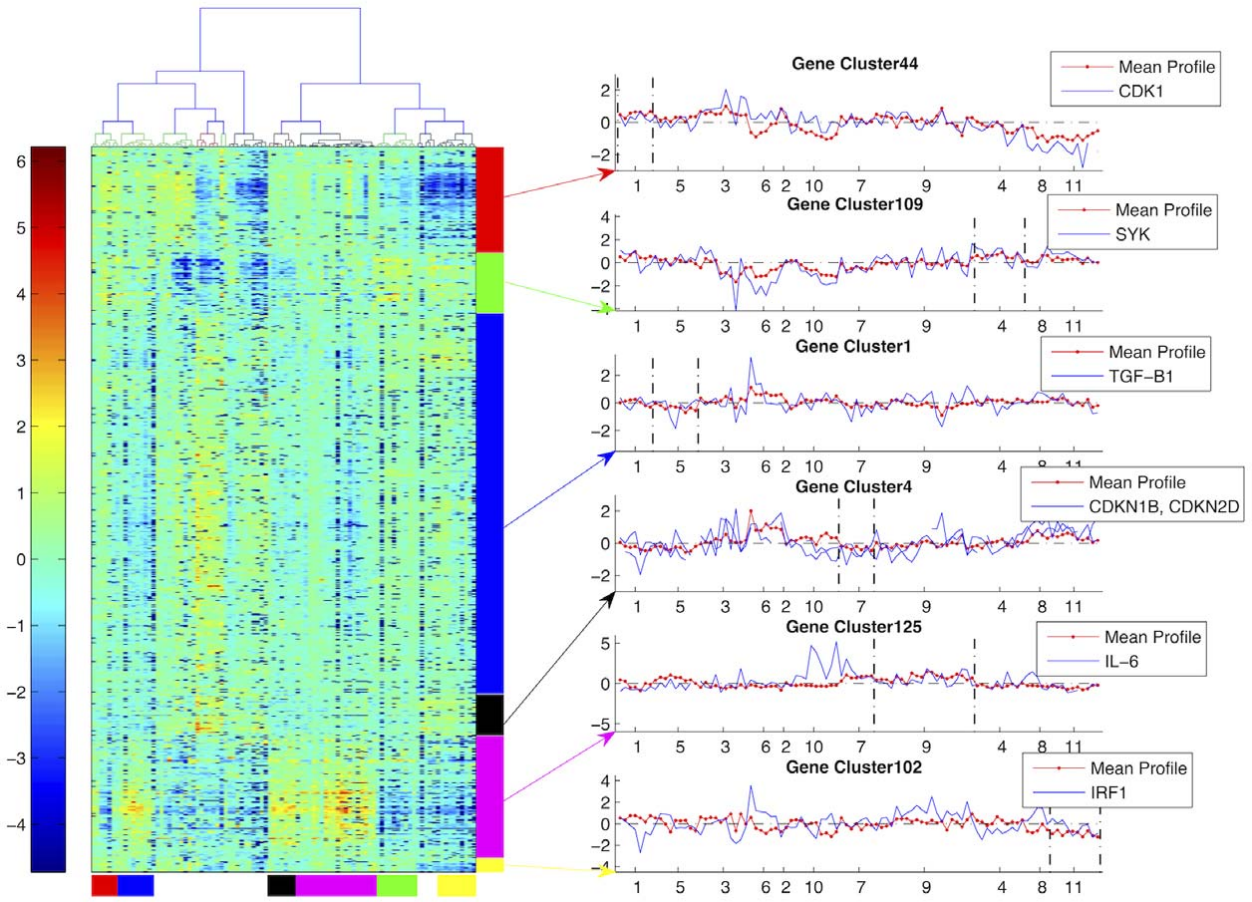
Expression profiles for six significant gene-clusters obtained by the DHBT method. *Left*: Heat map of gene expression profiles for the clusters of genes. Each row represents the expression profile from a clone, and each column represents a sample. The samples are organized according to the DBHT hierarchy as shown on the dendrogram on the top. Significant gene-clusters are highlighted with different colors as follows (from top to bottom, colours online): Red - gene-cluster ‘44’ (significant for sample-cluster ‘1’); Green - gene-cluster ‘109’ (significant for sample-cluster ‘4’); Blue - gene-cluster ‘1’ (significant for sample-cluster ‘5’); Black - gene-cluster ‘4’ (significant for sample-cluster ‘7’); Magenta - gene-cluster ‘125’ (significant sample-cluster ‘9’); Yellow - gene-cluster ‘102’ (significant for sample-cluster ‘11’). The same color scheme is used on the bottom of the heat-map to denote the corresponding sample-clusters. *Right*: Mean expression profile for each gene-cluster together with the expression profiles of note-worthy gene for each sample-cluster. The x-axes report the gene clusters. The boundaries of the relevant sample-cluster for each gene-cluster are indicated with the vertical dashed lines.

Let us now move a step further and use the DBHT technique to identify significant groups of genes that are of relevance for particular cancer samples. Indeed, an accurate identification of significant genes is crucial in treating the tumor cells as there are a large number of different genetic mechanisms from which these tumor cells originate, hence they require different treatments [54], [55]. We have therefore performed a two-way clustering: on the samples and genes simultaneously. In this way, we can cross-tabulate the samples against genes obtaining a simple and effective picture of significant gene expression patterns. Let us note that with conventional clustering techniques, the two-way clustering adds another dimension of complexity. Indeed, samples and gene expression profiles have different dimensions and scales and therefore it is necessary to tune the clustering parameters separately for each clustering way. On the other hand, the DBHT technique has no adjustable parameters and it is deterministic providing therefore a unique cross classification without any increase in complexity. The DBHT technique identifies 180 gene-clusters from which we have extracted 6 clusters which are significantly differentiating for sample-clusters associated to FL, CLL and DLBCL, accordingly with a p-value threshold of 0.01 with Bonferroni correction. The expression profiles of these significant gene-clusters are reported in Fig. 10. We have then validated functional significance of these gene-clusters by performing a gene-ontology (GO) analysis to identify significant GO terms for biological processes [56]. (See Supporting Information S1 for the statistical analysis methods and GO results.) Let us here report on some relevant genes, from each of the 6 significant gene-clusters, selected by choosing the most frequently appearing genes in the GO terms. Interestingly, these genes reveal some of biologically significant mechanisms that regulate growth of tumor cells, and that affect survival of respective lymphoma malignancy. In particular:

- Gene cluster ‘44’ (significant for sample-cluster ‘1’): This gene-cluster is up-regulated for sample-cluster ‘1’ in comparison to the expressions in other sample-clusters associated to lymphoma. Significantly, one of its key genes is CDK1, which is a key player in cell cycle. It has been indicated that over-expression of CDK1 is common in DLBCL cancer types, and it is therefore a potential therapeutic target [57].
- Gene cluster ‘4’ (significant for sample-cluster ‘4’): This gene-cluster particularly expresses for sample-cluster ‘4’, which consists mostly of FL samples. Among the genes in this gene-cluster there is SYK which -indeed- has been indicated as a promising target gene for antitumor therapy for treating FL, where inhibition of SYK expression increases the chance of survival [58].
- Gene cluster ‘1’ (significant for sample-cluster ‘5’): Gene cluster 1 is particularly down-regulated for sample-cluster ‘5’. This gene-cluster contains TGF-B1 which is a well-known transcription factor to regulate proliferation, in particular a negative regulator of B-cell lymphoma which induces apoptosis of the tumor cells via NF-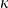
B/Rel activity [59]. This suggests that suppression of the tumor cells by TGF-B1 would be lessened in sample-cluster ‘5’ due to the down-regulation, and this may contribute to the decreased chance of survival observed in sample-cluster ‘5’ in comparison to that of sample-cluster ‘1’.
- Gene cluster ‘4’ (significant for sample-cluster ‘7’): This gene-cluster is slightly down-regulated for sample-cluster ‘7’, and GO analysis extracts two genes, CDKN1B/p27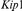 and CDKN2D/p19, which are key tumor suppressor genes for aggresive neoplasms [60], [61]. The inhibited tumor suppressive role of these genes might have led to aggressive growth of tumor cells suggesting a plausible explanation for the poorest survival rate, observed for sample-cluster ‘7’, with respect to the other DLBCL sample-clusters (see Table 1). Indeed, it has been suggested that p27 is associated to lymphomagenesis through Skp2 [61] and Skp2 has been indicated as an independent marker to predict survival outcome in DLBCL [61], [62].
- Gene cluster ‘125’ (significant for sample-cluster ‘9’): This gene-cluster shows distinct up-regulation pattern for sample cluster ‘9’, and it includes an interesting gene ‘IL-6’. IL-6 is known to be a central target gene in a synergistic crosstalk between NF-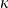
B and JAK/STAT pathway, which is a unique feature for some DLBCL [55]. It is suggested that, these have implications for targeted therapies by blocking STAT3 expression, a gene that is activated by IL-6 [55], [63].
- Gene cluster ‘102’ (significant for sample-cluster ‘11’): This gene-cluster particularly down-regulates the CLL sample-cluster among all lymphoma-related clusters. Though it does not indicate a particularly significant GO term (see Supporting Information S1), it includes a number of genes related to regulating tumor cell growth for CLL (see Supporting Information S1 for the list of genes). Among these genes, let us note IRF1, which is a well-known mediator for cell fate by facilitating apoptosis, and it is also a tumor suppressor [64]. As the expression of IRF1 is slightly down-regulated, we suspect that this may contribute to the growth of CLL tumor cells.

In conclusion let us stress that these results strongly indicate that the DBHT technique can detect relevant differentiations and aggregations in both cancer-samples and gene-clones revealing important relations that can be used for diagnosis, for prognosis and for treatment of these human cancers.

## Discussion

In summary, we have introduced a novel approach, the DBHT technique, to extract cluster structure and to detect hierarchical organization in complex data-sets. This approach is based on the study of the properties of topologically embedded graphs built from a similarity measure. The DBHT technique is deterministic, it requires no a-priori parameters and it does not need any expert supervision. We have shown that the DBHT technique can successfully retrieve the clustering and hierarchical structure both from artificial data-sets and from different kinds of real data-sets outperforming in several cases other established methods. The application of the DBHT technique to a referential gene-expression dataset [44] shows that this method can be successfully used in differentiating patients with different cancer subtypes from gene-expression data. In particular, we have correctly retrieved the differentiation into distinct clusters associated with cancer subtypes (FL, CL and DLBCL) along with a meaningful hierarchical structure. The DBHT technique provides a meaningful differentiation of the DLBCL cancer samples into four distinct clusters which turn out to correspond to different survival rates. The application of the DHBT clustering technique over the gene-clones identifies new groups of genes that play a relevant role in the differentiation of the cancer subtypes, and possibly in relevant genetic pathways which control survival/proliferation of the tumor cells. Differently from [44] which indicates GCB- and ABC-like DLBCL classification under thorough supervision with biological expertise, we have found instead, in a completely un-supervised manner, four subtypes of DLBCL with different expression signatures that differentiate significantly in their genetic mechanisms and biological features resulting in well distinct survival rates, hence providing a new perspective. It should be stressed that the DBHT technique is addressing the problem of data clustering and hierarchical study from a different perspective with respect to other approaches commonly used in the literature. It therefore provides an important alternative support in a field where the sensitivity of the results to the kind of approach is often crucial. The DBHT technique can be extended to more complex measures of dependency which may be also asymmetric. In our graph theoretic approach this can be handled by constructing topologically embedded directed graphs. Another extension may concern the use of graph-embedding on surfaces of genus larger than zero that will provide more complex networks and a richer data filtering [17].

## Supporting Information

Supporting Information S1 The file *PaperSupporting_ver230112_PLoSOne.pdf* contains additional information to the manuscript explaining methods, procedures and results in further details. It consists of 16 pages, 3 tables and 11 figures.
(PDF)

Supporting Information S2 The file *DHBT_codesAndData.zip* is a compressed achieve file containing the matlab code *DBHT.m* to compute the DBHT clusters and hierarchies, this code calls 8 other functions: *BubbleCluster8.m, CliqHierarchyTree2.m, BubbleCluster8.m, clique3.m, cRand1.m, DirectHb.m, doPMFG.m, DrawPMFG.m*. The achieve also contains the code *iris_demo.m* and the data *matlab_iris_demo.mat* which can be used to reproduce Fig. 5(a). Demo code and dataset to reproduce Fig. 9 are instead: *ymphoma_demo.m, matlab_DLBCL_demo.mat*. The *ReadMe.tex* file explains code usage and installation.
(ZIP)
