# Supplementary material for: Hierarchical Information Clustering by Means of Topologically Embedded Graphs
Source: PLoS One. 2012 Mar 9;7(3):e31929. doi: 10.1371/journal.pone.0031929 (PMC3302882; doi:10.1371/journal.pone.0031929)
Supplement: Supporting Information S1 — The file PaperSupporting_ver230112_PLoSOne.pdf contains additional information to the manuscript explaining methods, procedures and results in further details. It consists of 16 pages, 3 tables and 11 figures. (PDF) [file pone.0031929.s001.pdf]

Supporting information for: Hierarchical information clustering by means of topologically embedded graphs

Won-Min Song[1], T. Di Matteo[1,2], Tomaso Aste[1,3]

1 Applied Mathematics, Research School of Physics and Engineering, The Australian National University, Canberra ACT 0200, Australia.
2 Department of Mathematics, King's College London, London, WC2R 2LS, UK.
3 School of Physical Sciences, University of Kent, UK.
∗ E-mail corresponding author: tomaso.aste@anu.edu.au

S.1 Artificial data with a clustering structure

S.1.1 Preparation

By using a multivariate Gaussian generator (MVG) and a multivariate Log-Normal generator [see: Wang SS (2004) Casualty actuarial society proc. LXXXV] we have produced several synthetic time series which approximate a given correlation structure R^*. Specifically, we have generated N stochastic time series $y_i(t)$ of length T $(i = 1...N,\ t = 1...T)$ with zero mean and Pearson's cross-correlation matrix R that approximates R^*. As for the starting correlation structures R^*, we have used block diagonal matrices where the blocks are the artificial correlated clusters. The matrix R^* has zero inter-cluster correlations ρ^{ou*} and large intra-cluster correlations ρ^{in*} within the diagonal blocks. To this pre-defined cluster structure, we added a number N_{ran} of random correlations unrelated to the clusters. We have chosen $T = 10 \times N$ and we added a noise term $\eta_i(t)$ obtaining a new set of dataseries

$$y'_i(t) = y_i(t) + c\sigma_i\eta_i(t) \quad , \tag{S.1}$$

where $\sigma_i = \sqrt{\langle y_i^2\rangle - \langle y_i\rangle^2}$ is the standard deviation of $y_i(t)$ and c is a constant used to tune the relative amplitude of noise. We have used a Normally distributed noise with probability distribution function $p(\eta) \propto \exp(-\eta^2/2)$ and a log-Normally distributed noise with probability distribution function $p(\eta) \propto \exp(-\log(\eta)^2/2)$. We have varied the relative amplitude of noise c from 0 to 7 with constant intra-cluster correlation in R^* at $\rho^{in*} = 0.9$. We also have used power-law distributed noise, with probability distribution function $p(\eta) \propto 1/\eta^{\alpha+1}$. Specifically, this noise was numerically generated by using $\eta(t) = \pm|\eta^{un}(t)|^{(-1/\alpha)}$, where $\eta^{un}(t)$ is a uniformly distributed noise in $(0, 1]$ and the sign in front is chosen at random for each t with probability 50%. In this case, we have varied the relative amplitude of noise c from 0 to 0.8 with exponent $\alpha = 1.5$ and constant intra-cluster correlation $\rho^{in*} = 0.9$. We also have varied the exponent α between 1 to 3 keeping $c = 0.1$ and $\rho^{in*} = 0.9$. Examples of the obtained correlation matrices are reported in Fig.S.1 for the MVG and Fig.S.2 for the log-normal multivariate generator.

All these different manipulations produce a similar effect where by increasing the amplitude of noise or by decreasing the exponent or by reducing ρ^{in*}, the Pearson's cross-correlation matrix R passes from a very well defined structure close to R^* to a blurred structure where the average intra-cluster correlation ($\langle\rho^{in}\rangle$) becomes smaller and finally it becomes equal to the average inter-cluster correlation ($\langle\rho^{ou}\rangle$) and no correlation structure can be any longer observed.

In summary, the simulated data were generated by combining the following possibilities.

- Partitions:
  - Regular Partitions (all clusters of the same size),

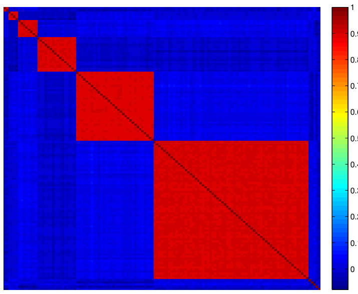

(a) Gaussian Noise, $c = 0.2$, $\rho^{in*} = 0.9$, $\rho^{ou*} = 0$, $N_{ran} = 5$.

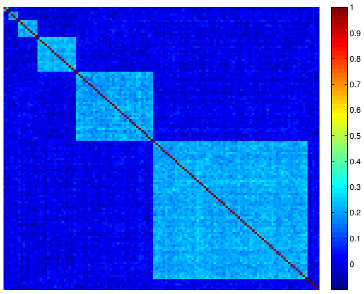

(b) Gaussian Noise, $c = 1.8$, $\rho^{in*} = 0.9$, $\rho^{ou*} = 0$, $N_{ran} = 5$.

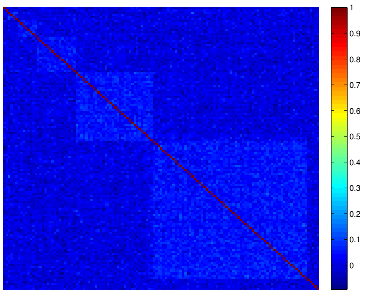

(c) Gaussian Noise, $c = 4.2$, $\rho^{in*} = 0.9$, $\rho^{ou*} = 0$, $N_{ran} = 5$.

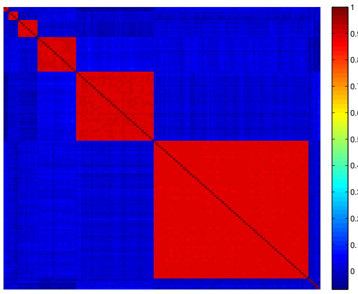

(d) Log-normal Noise, $c = 0.2$, $\rho^{in*} = 0.9$, $\rho^{ou*} = 0$, $N_{ran} = 5$.

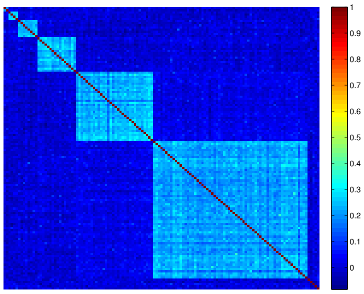

(e) Log-normal Noise, $c = 0.8$, $\rho^{in*} = 0.9$, $\rho^{ou*} = 0$, $N_{ran} = 5$.

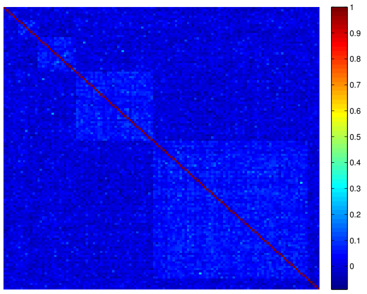

(f) Log-normal Noise, $c = 2$, $\rho^{in*} = 0.9$, $\rho^{ou*} = 0$, $N_{ran} = 5$.

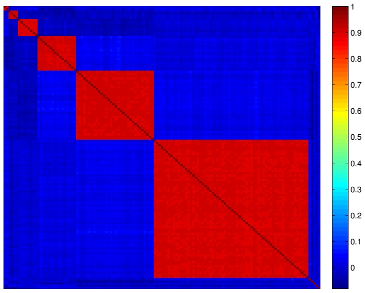

(g) Power-law Noise, $c = 0.05$, $\rho^{in*} = 0.9$, $\rho^{ou*} = 0$, $\alpha = 1.5$, $N_{ran} = 5$.

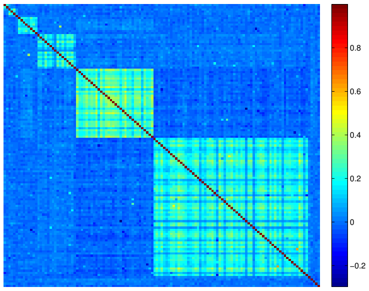

(h) Power-law Noise, $c = 0.25$, $\rho^{in*} = 0.9$, $\rho^{ou*} = 0$, $\alpha = 1.5$, $N_{ran} = 5$.

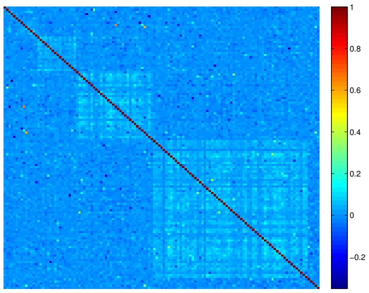

(i) Power-law Noise, $c = 0.65$, $\rho^{in*} = 0.9$, $\rho^{ou*} = 0$, $\alpha = 1.5$, $N_{ran} = 5$.

Figure S.1. Visualization of correlation matrices of synthetic data sets generated from MVG with partition of cluster sizes 4,8,16,32 and 64 where relative noise amplitude c has been varied to change the resolution of clustering structure. The parameters are specified underneath each figure. The first row adjusts c for Gaussian noises, the second adjusts for log-normal noises, and the third adjusts for Power-law noises with $\alpha = 1.5$.

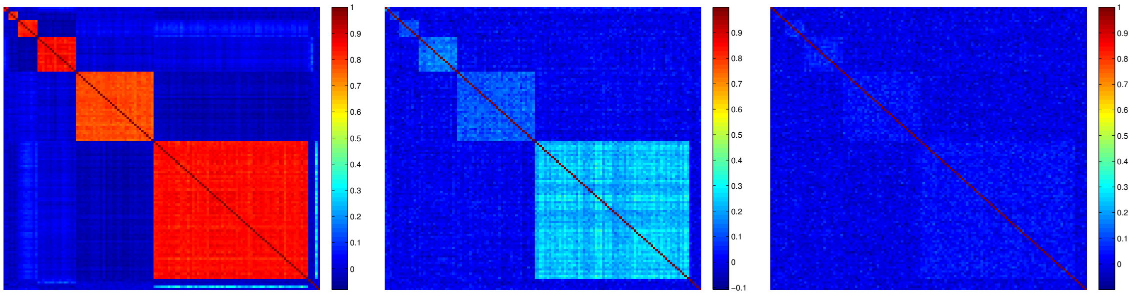

(a) Gaussian Noise, $c = 0.3$, $\rho^{in*} = 0.9$, $\rho^{ou*} = 0$, $N_{ran} = 5$.

(b) Gaussian Noise, $c = 2.1$, $\rho^{in*} = 0.9$, $\rho^{ou*} = 0$, $N_{ran} = 5$.

(c) Gaussian Noise, $c = 5.1$, $\rho^{in*} = 0.9$, $\rho^{ou*} = 0$, $N_{ran} = 5$.

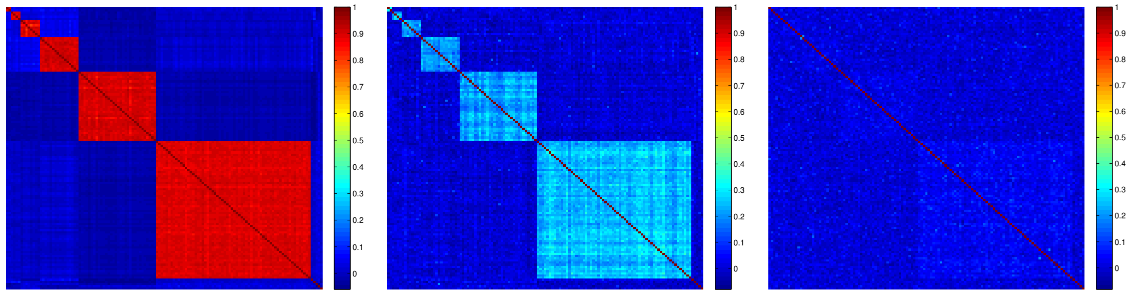

(d) Log-normal Noise, $c = 0.2$, $\rho^{in*} = 0.9$, $\rho^{ou*} = 0$, $N_{ran} = 5$.

(e) Log-normal Noise, $c = 0.8$, $\rho^{in*} = 0.9$, $\rho^{ou*} = 0$, $N_{ran} = 5$.

(f) Log-normal Noise, $c = 2.8$, $\rho^{in*} = 0.9$, $\rho^{ou*} = 0$, $N_{ran} = 5$.

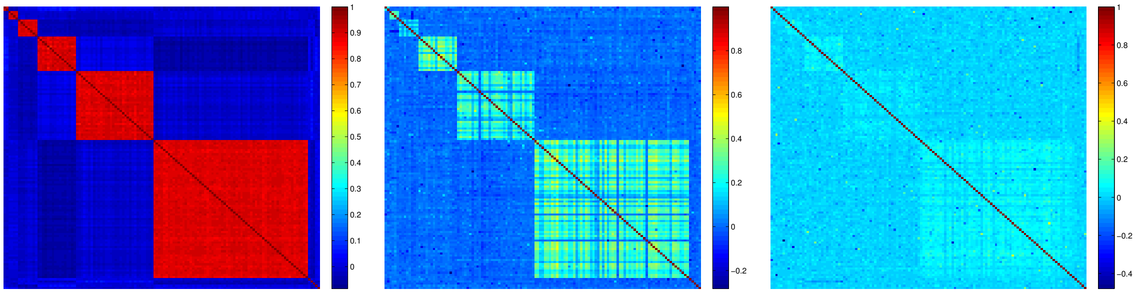

(g) Power-law Noise, $c = 0.05$, $\rho^{in*} = 0.9$, $\rho^{ou*} = 0$, $\alpha = 1.5$, $N_{ran} = 5$.

(h) Power-law Noise, $c = 0.25$, $\rho^{in*} = 0.9$, $\rho^{ou*} = 0$, $\alpha = 1.5$, $N_{ran} = 5$.

(i) Power-law Noise, $c = 0.65$, $\rho^{in*} = 0.9$, $\rho^{ou*} = 0$, $\alpha = 1.5$, $N_{ran} = 5$.

Figure S.2. Visualization of correlation matrices of synthetic data sets generated from log-normal multivariates with partition of cluster sizes 4, 8, 16, 32 and 64 where relative noise amplitude c has been varied to change the resolution of clustering structure. The parameters are specified underneath each figure. The first row adjusts c for Gaussian noises, the second adjusts for log-normal noises, and the third adjusts for Power-law noises with $\alpha = 1.5$.

  - Irregular partitions (clusters with different sizes).

- Type of multivariate random variables:
  - Multivariate Gaussian Distribution;
  - Multivariate Log-normal Distribution.
- Type of perturbation noises:
  - Univariate Gaussian Distribution;
  - Univariate Log-normal Distribution;
  - Univariate Power-law Distribution.
- Relative noise amplitude c.
- Random background elements N_{ran}.

S.1.2 Comparison with different clustering methods

Figure S.3 shows the performance curves evaluated via adjusted Rand index for simulated data with multivariate Gaussian distribution and Fig.S.4 shows the performance curves for simulated data with multivariate Log-normal distribution. The results for a wide range of $dR > 0.1$ for a broad set of combinations show that DBHT clustering outperforms the other clustering techniques except for Qcut which performs similarly to the DBHT. However, Fig.S.5 shows that the DBHT clustering can outperform also Qcut for both Gaussian and Log-normally simulated data when an extreme cluster size differentiation is present. Specifically, in Fig.S.5, there is a structure of eight small clusters of size 5 elements and one big cluster of 64 elements, and large number of random background elements ($N_{ran} = 25$). Let us stress that the performance curves in Fig.S.5 demonstrate that DBHT clustering is the only technique which delivers consistent and quality clustering outcomes in spite of the severe conditions applied.

S.2 Artificial data with a hierarchical Structure

S.2.1 Preparation

In order to test the DBHT technique for the detection of the hierarchical structure, we have generated input matrices R^* that are organized in a nested block-diagonal structure where block of small sizes are placed inside blocks of lager sizes. In particular, we looked at regular partitions of 16 'small' clusters containing 16 elements each with $\rho_1^{in*} = 0.95$. These small clusters are merged to 'medium' clusters with $\rho_2^{in*} = 0.8$, and further merged to 'big' clusters with $\rho_2^{in*} = 0.7$. Finally, all clusters are merged to a single cluster with $\rho^{ou*} = 0.15$. Similarly, we looked at irregular partitions with clusters of scaling sizes containing, 4, 8, 16, 32 and 64 elements each, and the structures of small, medium, and big clusters were embedded by consecutively merging with $\rho_1^{in*}, \rho_2^{in*}, \rho_3^{in*}$ and ρ^{ou*}.

S.2.2 Comparison with different linkage methods

We have simulated 30 different sets of multivariate Gaussian data series of length $T = 10 \times N$ by using nested hierarchical block-diagonal input matrices R^*. An example of R^* is provided in Fig.S.6(a) (same as Fig.2(a) in the paper). We have tested the capability of the DBHT method to recognize hierarchies by moving through the different hierarchical levels varying the number of clusters from only one at the top hierarchy to the number of elements at the lowest hierarchy. At each number of clusters we have measured the adjusted Rand index with respect to the 'large', 'medium' and 'small' partitions. Figs.S.7(b-d) show

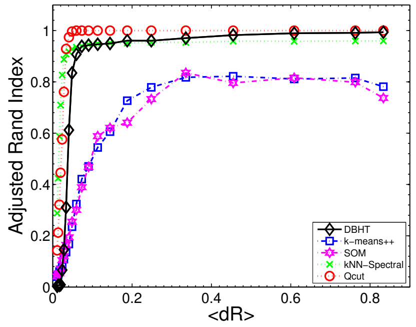

(a) Gaussian data with Gaussian noise and regular Partition.

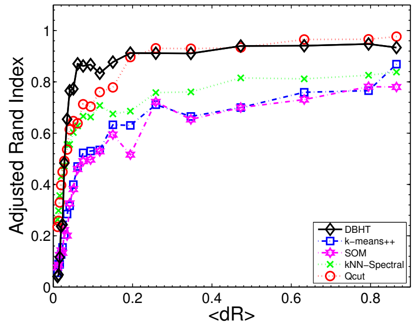

(b) Gaussian data with Gaussian noise and irregular Partition.

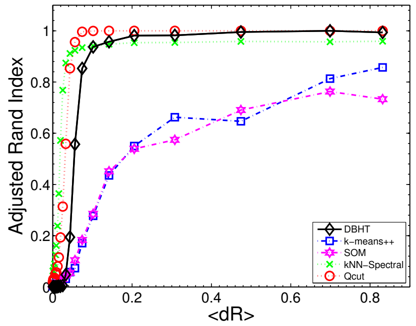

(c) Gaussian data with lognormal noise and regular Partition.

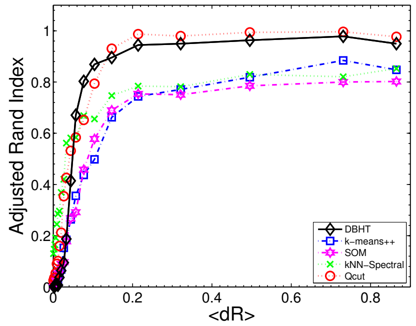

(d) Gaussian data with lognormal noise and irregular Partition.

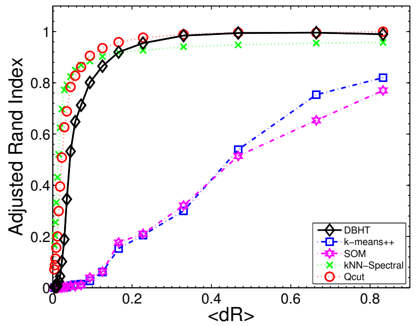

(e) Gaussian data with power-law noise and regular Partition.

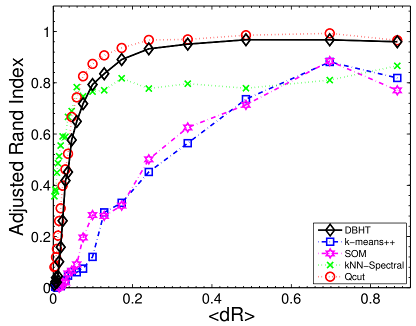

(f) Gaussian data with power-law noise and irregular Partition.

Figure S.3. Adjusted Rand index for various data sets simulated via Gaussian (Normal) distribution with $\rho^{in*} = 0.9, \rho^{ou*} = 0$ and $N_{ran} = 5$. For each value of c (see Eq.S.1), 30 data sets were generated in order to get stable statistics for $< dR >$ and adjusted Rand score.

the average adjusted Rand index and the standard deviations over the 30 sets of synthetic data obtained by using the DBHT method, the average linkage method and the complete linkage method. One can observe in Fig.S.7(b) that all three methods successfully detect the 4 large clusters retrieving adjusted Rand index near to unity. At following hierarchical levels only the DBHT method consistently retrieves the maximum value for the adjusted Rand index respectively at the hierarchical partitions with 8 and 16 clusters. Conversely, the other two methods achieve lower maximal values of the adjusted Rand index at

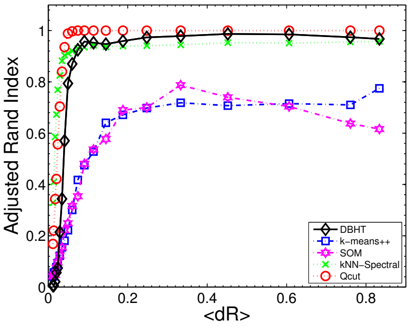

(a) Lornogmal data with Gaussian noise and regular Partition.

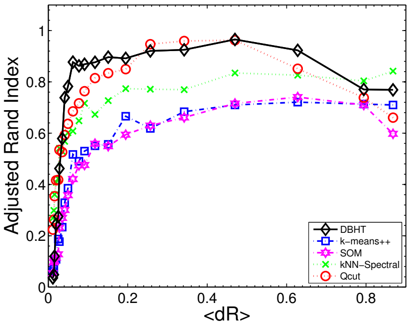

(b) Lognormal data with Gaussian noise and irregular Partition.

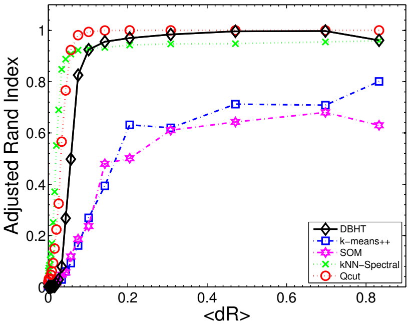

(c) Lognormal data with lognormal noise and regular Partition.

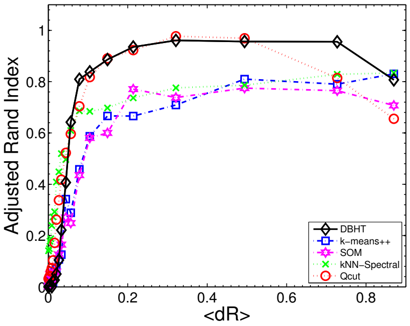

(d) Lognormal data with lognormal noise and irregular Partition.

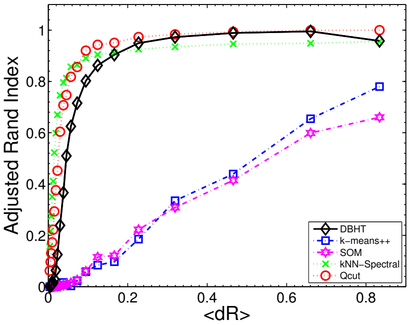

(e) Lognormal data with power-law noise and regular Partition.

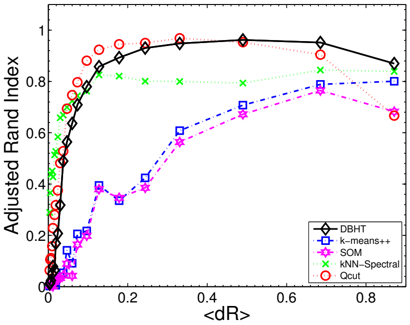

(f) Lognormal data with power-law noise and irregular Partition.

Figure S.4. Adjusted Rand index for various data sets simulated via Log-normal distribution with $\rho^{in*} = 0.9, \rho^{ou*} = 0$ and $N_{ran} = 5$. For each value of c (see Eq.S.1), 30 data sets were generated in order to get stable statistics for $< dR >$ and adjusted Rand score.

a larger number of clusters inconsistent with the sizes of the synthetic data structure. We have tested other partitions and different levels of noise verifying that the DBHT method is consistently delivering good performances in comparison with the other established methods. An example, by using power law noise and clusters of scaling sizes respectively of 4, 8, 16, 32 and 64 elements is reported in Fig.S.8(a). The dendrograms for the DBHT, and the average linkage and the complete linkage methods are respectively reported in Figs.S.8(b,c,d). The comparison between the adjusted Rand indexes is reported in Fig.S.9.

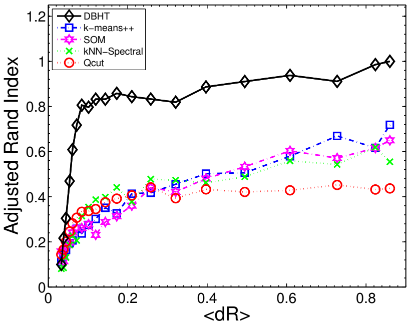

(a) Gaussian Data with Gaussian Noise.

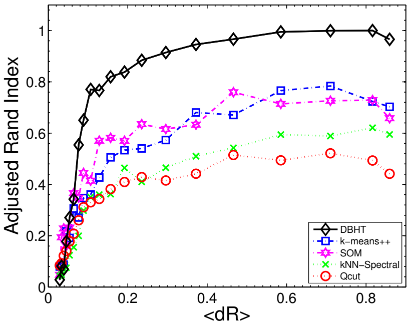

(b) Gaussian Data with Lognormal Noise.

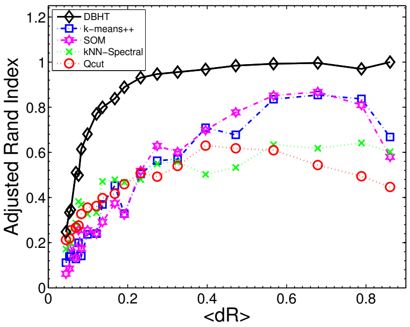

(c) Gaussian Data with Power-law Noise.

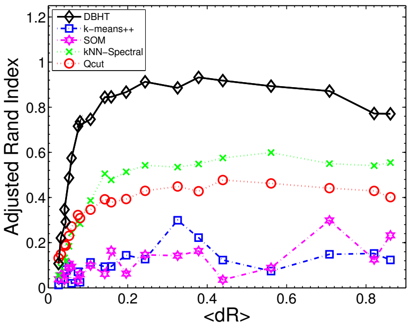

(d) Log-normal Data with Gaussian Noise.

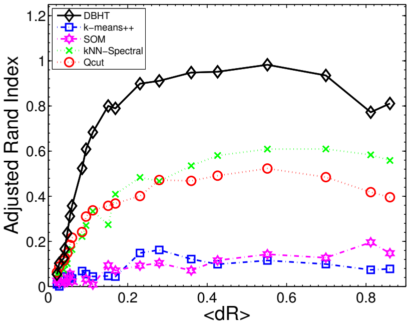

(e) Log-normal Data with Lognormal Noise.

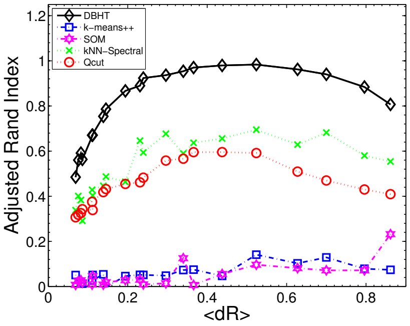

(f) Log-normal Data with Power-law Noise.

Figure S.5. Adjusted Rand index for various data sets simulated via Gaussian and Log-normal distribution with $\rho^{in*} = 0.9, \rho^{ou*} = 0$ and $N_{ran} = 25$. This case refers to a cluster structure with eight clusters of size 5 elements, and one cluster of size 64 elements. For each value of c (see Eq.S.1), 30 data sets were generated in order to get stable statistics for $< dR >$ and adjusted Rand score. Figure (a) and (f) are the same of Fig.1 in the paper and are here reported for completeness and for an easier comparison.

One can see that, also in this case, the DBHT technique consistently outperforms the linkage methods.

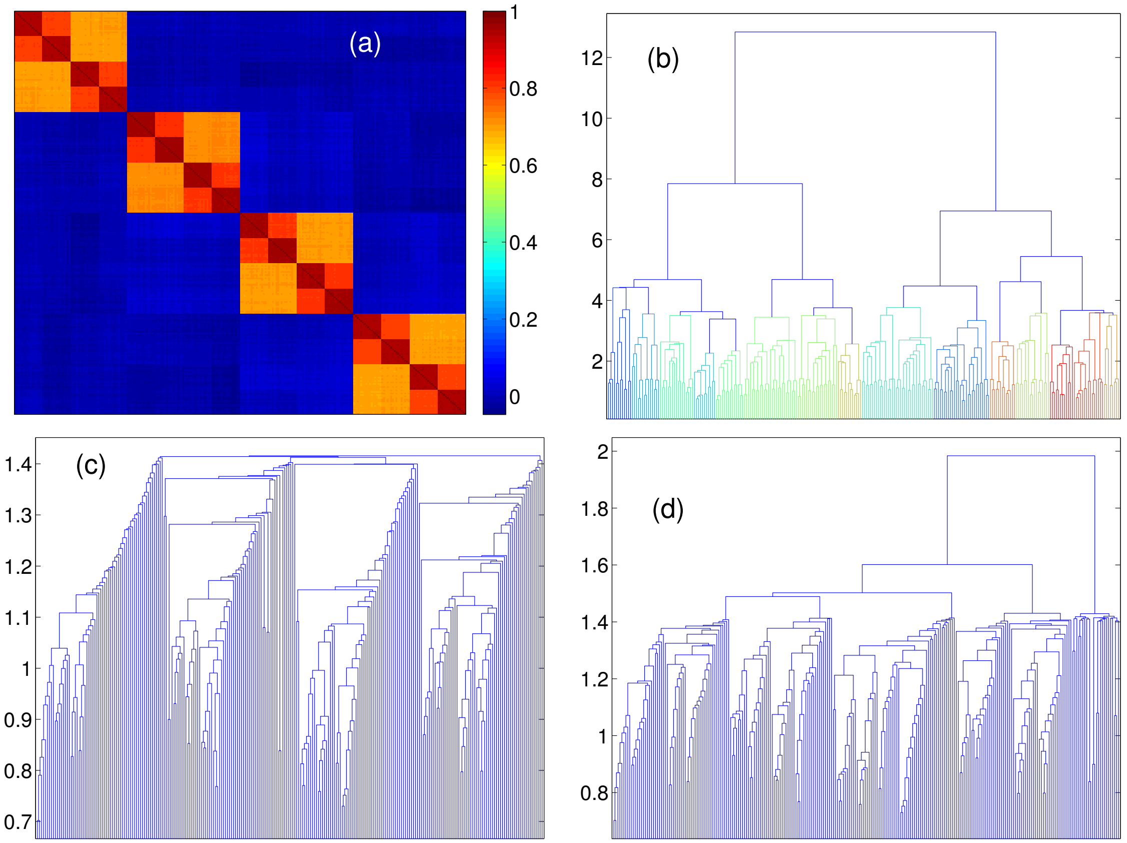

Figure S.6. Hierarchical clustering for uniform partition with a power law noise with exponent $\alpha = 1.1$ and noise level $c = 0.03$ **(a)** Correlation template R^* for a synthetic data structure with uniform sizes of 16 elements each. **(b)** Dendrogram associated with the DBHT hierarchical structure. **(c)** Dendrogram associated with the Average linkage. **(d)** Dendrogram associated with the Complete linkage.

S.3 Lymphoma data analysis

S.3.1 Emergence of GCB-like and ABC-like Patterns on PMFG

Here, we report how the GCB-like and ABC-like classification of DLBCL subtypes naturally emerges in the PMFG. This is shown in Fig.S.10 where we can observe that ABC-like DLBCLs are dominant on the top of PMFG, and mainly occupy sample-cluster '7' and '9'. On the other hand, GCB-like DLBCLs are dominant on the center of PMFG, and mainly occupy sample-cluster '1', '5' and '7'. Among the sample-clusters associated with DLBCL, sample-cluster '1' and '5' are distinctively characterized by GCB-like DLBCL, sample cluster '9' is characterized by ABC-like DLBCL. Interestingly, sample cluster '1' and '5' indicate a further sub-classification of GCB-like DLBCL, and yet show superior survival rates than sample clusters associated ABC-like DLBCL, a more fatal subtype indicated by Alizadeh *et al* 2000 than GCB-like DLBCL (See Table 1 in the main paper). Furthermore, sample-cluster '7' is a mixture of these two subtypes, and it yet shows much worse survival rates than sample-cluster '9' in which is present a much larger portion of ABC-like DLBCL (See Table 1 in the main paper). This clearly shows that the DBHT clustering indicates further meaningful subtypes of DLBCLs with respect to the GCB-/ABC-like classification of Alizadeh *et al* 2000.

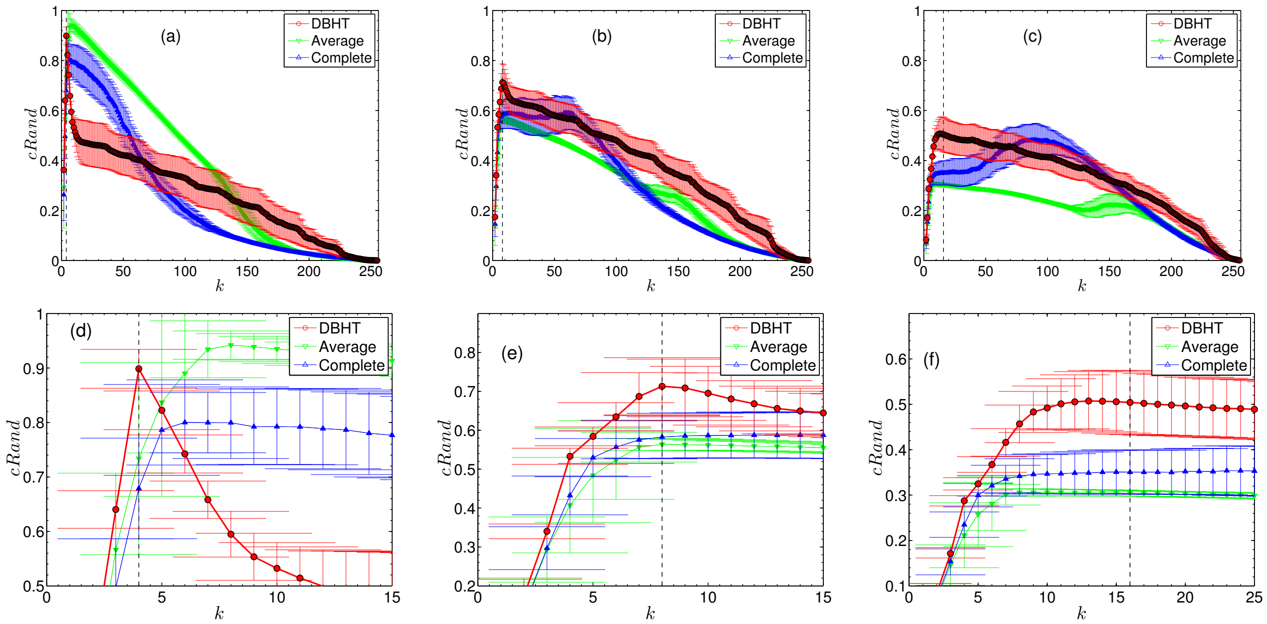

Figure S.7. Adjusted Rand index for the comparison between the synthetic partition in Fig.S.6(a) and the partitions retrieved by cutting the dendrograms from our DBHT clustering method at various numbers of clusters. **(a)** Comparison between the synthetic partition with the 4 large clusters and the partitions from DBHT, average linkage and complete linkage. **(b)** Comparison between the synthetic partition with the 8 medium clusters and the partitions from DBHT, average linkage and complete linkage. **(c)** Comparison between the synthetic partition with the 16 small clusters and the partitions from DBHT, average linkage and complete linkage. **(d),(e),(f)** Details of the upper figures showing the region where the DBHT has the maximum. The plots report average values over a set of the 30 trials, the error bars are the standard deviations.

S.3.2 Analysis of significant gene-clusters for sample-clusters

In order to look for significant gene-clusters which distinguish each sample-cluster, we have performed a series of statistical analysis on the gene-clusters of the data found by DBHT clustering. Specifically, we have performed a combination of differential expression and enrichment analyses. Firstly, for a given sample-cluster, we have looked for a set of differentially expressed gene-profiles for a given cut-off p-value. Then we have calculated enrichment statistics for each gene-cluster by asking whether this cluster significantly enriches for the differentially expressed profiles. By varying the cut-off p-values, we have identified the most significant gene-cluster for the particular sample-cluster by choosing the gene-cluster that remains significantly enriched for the smallest cut-off. In order to identify differentially expressed profiles for each cut-off p-value, we have performed non-parametric Kruskal-Wallis one-way ANOVA test. The enrichment statistics has been evaluated by using the hypergeometric test with significance level of p-value 0.05, where the p-values were adjusted by Bonferroni correction. Fig.S.11 reports the smallest cut-off p-values for each gene-cluster, for each sample-cluster. The list of labels for the most significant gene-clusters is shown in Table 1. Except for sample-cluster '2' and '6', each sample-cluster is assigned to a unique gene-cluster. For what concerns sample-cluster '2' this is most likely due to the small cluster size. Instead, we note that sample-cluster '6' corresponds to a collection of T Cell samples, and we suspect that the emergence of multiple significant gene-clusters is due to the broad spectrum of T cells in the physiology of lymphoma.

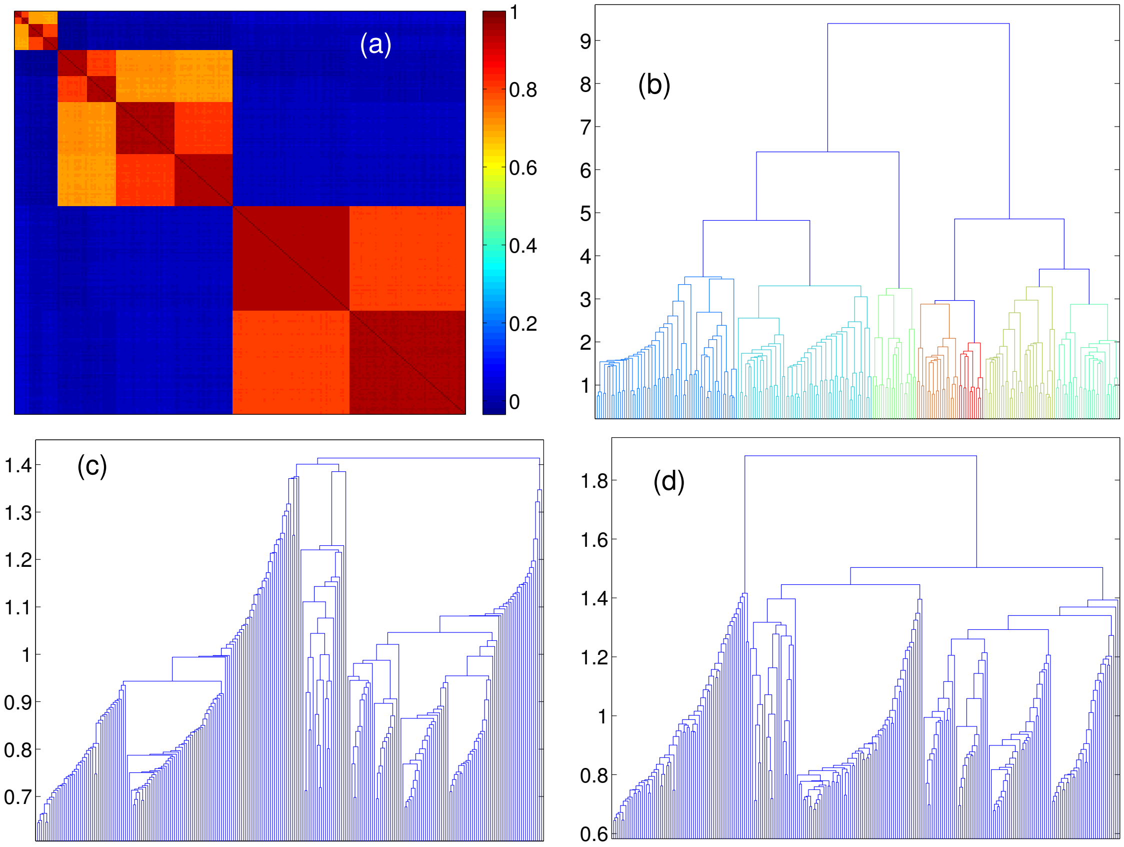

Figure S.8. **(a)** Correlation template R^* for a synthetic data structure with clusters with scaling sizes of 4, 8, 16, 32 and 64. **(b)** Dendrogram associated with the DBHT hierarchical structure. **(c)** Dendrogram associated with the Average linkage. **(d)** Dendrogram associated with the Complete linkage.

S.3.3 Gene Ontology analysis on significant gene-clusters

Among all significant gene-clusters, we have chosen a subset of gene-clusters which are associated to lymphoma malignancies, and we have performed Gene Ontology (GO) analysis on these gene-clusters in order to investigate associated biological processes. The analysis has been performed with significance level of p-value 0.05 on a plug-in software for Cytoscape, called BiNGO, and we applied Bonferroni correction. We have obtained a number of significant biological processes which are reported in Table 2. These biological processes indicate the underlying genetic mechanisms of which genes in the same gene-cluster share. For instance, gene-cluster '44' is associated to a large number of GO terms for cell cycles and cell cycle regulation. Indeed, this gene-cluster contains, for various phases, a key cell-cycle regulator CDK1 whose over-expression pattern is a characteristic feature of DLBCL as discussed in the main paper. On the other hand, none of the significant biological processes was captured by GO analysis for gene-cluster '102'. However, by no means, this cluster is un-significant for the sample-cluster. Indeed, as the enrichment analysis in Fig.S.11 suggests, gene-cluster '102' remained enriched for very low p-value $\sim 10^{-6}$, and it includes biologically significant genes for CLL such as IRF1 as reported in the main paper. In Table 3 we report the full list of clones for the gene-cluster '102'.

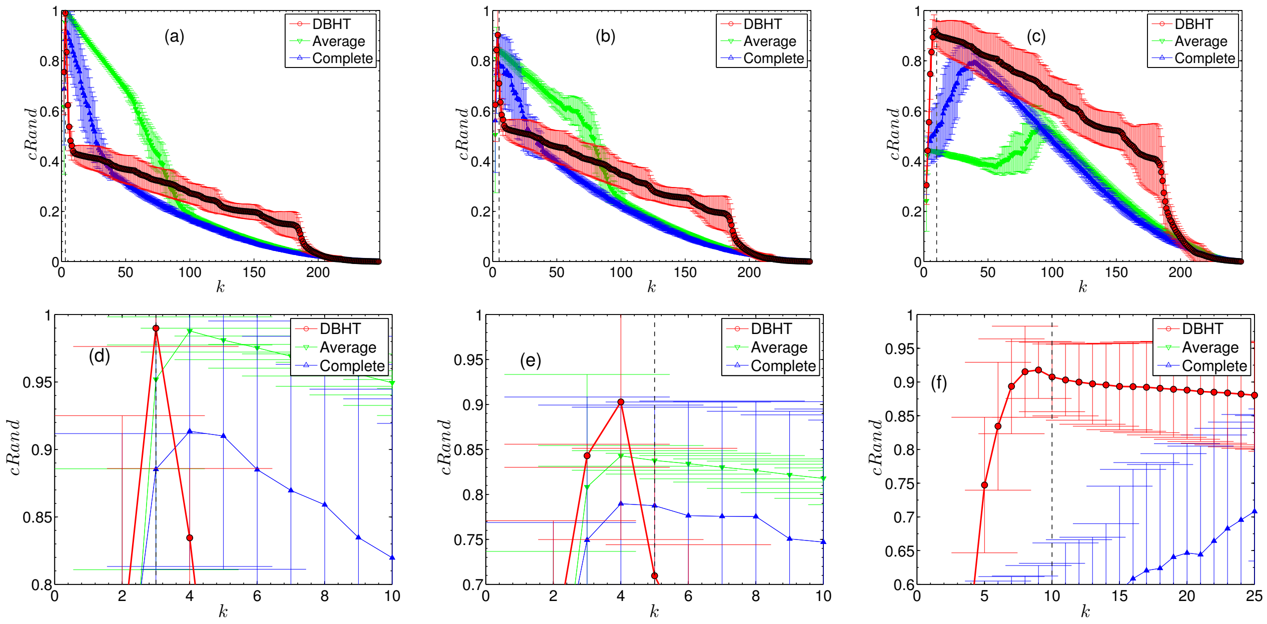

Figure S.9. Adjusted Rand index for the comparison between the synthetic partition in Fig.S.8(a) and the partitions retrieved by cutting the dendrogram from the DBHT clustering method at various number of clusters. **(a)** Comparison between DBHT clustering and the synthetic partition with the 2 'large' clusters. **(b)** Comparison between DBHT clustering and the synthetic partition with the 5 'medium' clusters. **(c)** Comparison between DBHT clustering and the synthetic partition with the 10 'small' clusters. **(d),(e),(f)** Details of the upper figures showing the region where the adjusted Rand index from DBHT has the maximum. The plots (b), (c) and (d) report average values over a set of the 30 trials, the error bars are the standard deviations.

S.4 Computational Complexity Analysis

Computationally expensive operations in DBHT algorithm include:

I) construction of PMFG;

II) construction of bubble hierarchical tree;

III) construction of DBHT hierarchy;

IV) computation of shortest path lengths.

S.4.1 Construction of PMFG

Construction of PMFG consists of two main steps: i) sorting the list of all pairs of vertices by the respective similarity values; ii) checking the planarity of the graph when a new edge is added. We have used the built-in command 'sort()' in MATLAB, which runs in $\mathcal{O}(n\log(n))$for n elements. Since the sorting takes place on the full list of $|V|(|V|-1)/2$ pairs, its time complexity is $\mathcal{O}(|V|^2\log(|V|))$.
In order to check the planarity, we have utilized Boyer-Myrvold algorithm. The algorithm runs in $\mathcal{O}(|V|)$ [1], therefore the worst case runtime is $\mathcal{O}(|V|^3)$ for $|V|(|V|-1)/2$ edges. In practice, the PMFG algorithm terminates well before it reaches the end of the sorted list of edges, and the growing planar graph contains

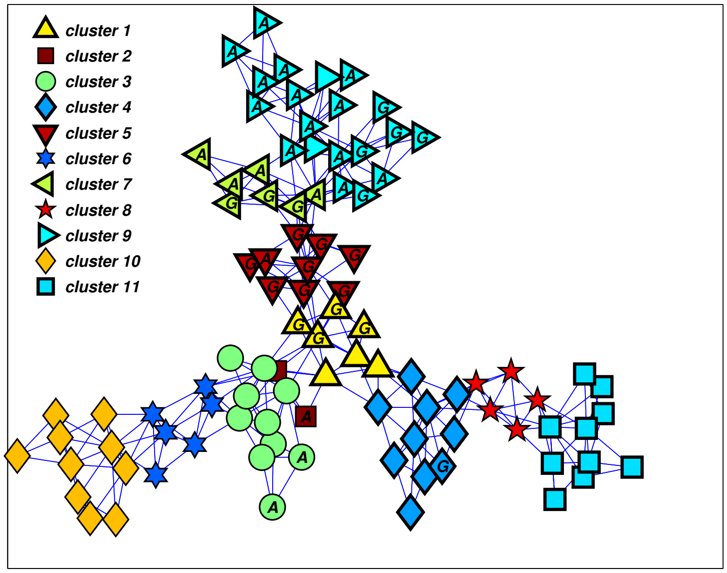

Figure S.10. Visualization on the PMFG of the GCB-like and ABC-like classifications as given by Alizadeh *et al* 2000. The labels inside the symbols correspond respectively to GCB-like DLBCL (G) and ABC-like DLBCL (A). The symbols are the same used to represent the sample clusters found by DBHT technique in Fig.4 in the main paper .

less vertices than $|V|$. Therefore, the overall time complexity for checking planarity of all edges in the PMFG follows $\mathcal{O}(|V|^{\alpha})$ with $\alpha < 3$.
We have tested the runtime of PMFG algorithm on MATLAB for a $|V| \times |V|$ Pearson's correlation matrix with a clustering structure made of two equal-sized clusters with $\rho^*_{in} = 0.3$, generated from multivariate Gaussian time series of $|V|$ elements of length $10|V|$. We have observed that the runtime scales with $\mathcal{O}(|V|^{\alpha})$ with $\alpha \sim 2.5$.

S.4.2 Construction of bubble hierarchical tree

The core of the construction of the bubble hierarchical tree consists of three main steps [2]: i) identifying all 3-cliques k_p; ii) finding their respective interior/exteriors $G_p^{in/ex}$; iii) checking the separating property. In order to identify all 3-cliques, we have utilized a simple search algorithm based on the detection of common neighbors of vertices linked by an edge. Specifically, we have implemented the following algorithm: (a) initialize the list of 3-cliques **CliqList**; (b) list the edges in the PMFG; (c) for each edge, look for the set of common neighbors and retain the set of respective 3-cliques; (d) for each 3-clique from (c), check if the 3-clique is present in the list **CliqList**, and add to **CliqList** if not. Utilizing sparsity of PMFG, (a) and (b) demand $\mathcal{O}(|V| + |V|^{\alpha})$ with $\alpha < 2$. Time complexity of step (c) depends on the number of 3-cliques in the PMFG, which is linear in $|V|$ [3]. Therefore, in the worst case, time complexity for checking presence of one 3-clique in **CliqList** is linear in $|V|$, and it gives the upper bound of the entire operation $\mathcal{O}(|V|^2)$.
Once **CliqList** is complete, we perform the searches for $G_p^{in/ex}$ for all 3-cliques to build the bubble hierarchy. Specifically, for each k_p, we do four operations: 1) remove k_p from G; 2) choose a random vertex v_o from G/k_p; 3) perform Breadth First Search (BFS) from v_o to identify interior/exterior of k_p; 4) repeat 1)-3) for all k_p. Steps 1 and 2 are computationally fast. Time complexity of step 3) depends

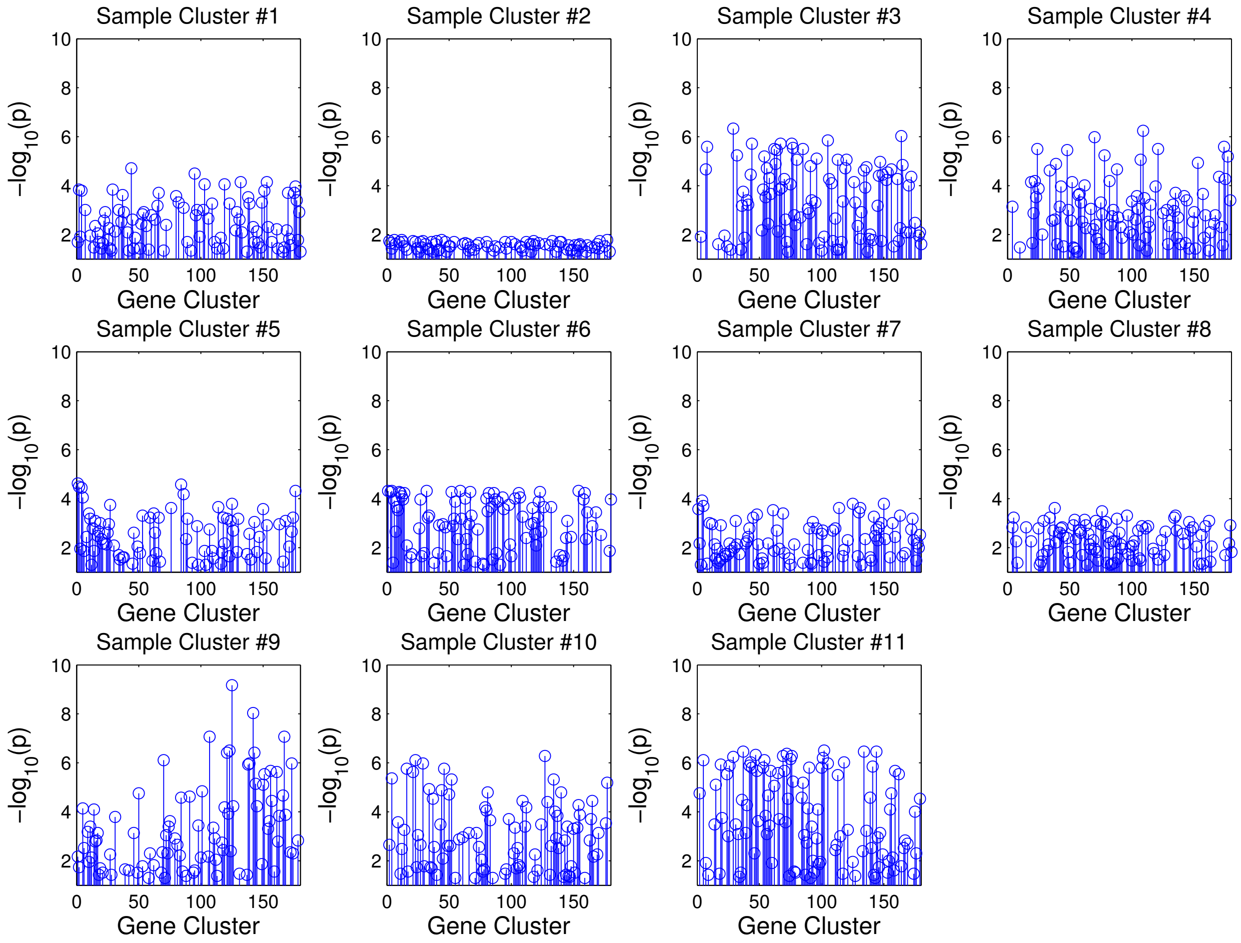

Figure S.11. Plot of cut-off p-value for Kruskal-Wallis one-way ANOVA test -vs- enriched gene-clusters. Circles represent the smallest cut-off p-value for individual gene-clusters.

on BFS, which is known to be $\mathcal{O}(|E'| + |V'|)$ where E' and V' are the sets of visited edges and vertices. Since some 3-cliques of PMFG are separating, it implies that BFS may explore a small portion of V, this gives an upper bound of $\mathcal{O}(|E| + |V|) = \mathcal{O}(|V|)$. Consequently, the time complexity for performing BFS for all 3-cliques is no larger than $\mathcal{O}(|V|^2)$.

Lastly, in step 4 the hierarchical tree is built by comparing the $(k_p \cup G_p^{in})$ for each k_p to all other $(k_q \cup G_q^{in})$ checking the relation $(k_p \cup G_p^{in}) \subseteq (k_q \cup G_q^{in})$ according to Ref. [2]. For a given pair of k_p and k_q, this requires to perform at most $|V|$ operations, to check interior membership of vertices. Repeating this for all k_p, it takes $\mathcal{O}(|V|^2)$ at most. Altogether, the worst running time of bubble hierarchy construction follows $\mathcal{O}(|V|^2)$.

In practice, the runtime is significantly lower in PMFG, because PMFG of a clustered data set tends to produce a large number of separating 3-cliques, and reduces the runtime to perform BFS. We have checked the empirical runtime to compute the bubble hierarchy in the PMFGs computed in Subsect. S.4.1, and it scales approximately with $\mathcal{O}(|V|)$.

Sample Cluster	Gene Cluster
1	44
2	6,12,44,177
3	29
4	109
5	1
6	1,4,32,59,154
7	4
8	38
9	125
10	127
11	102

Table 1. List of most significant gene-clusters for the sample-clusters. Sample clusters in bold italic font correspond to the clusters associated to lymphoma malignancies.

S.4.3 Construction of DBHT hierarchy

In the tailored method employed for DBHT hierarchy the bubble hierarchy, and clustering information provide sub-divisions within clusters and distance metric needs to be computed and sorted only within each sub-division and within/between clusters. This speeds up considerably the runtime of our algorithm with respect to a naive complete linkage which takes, at least, $\mathcal{O}(|V|^2)$ mainly due to the computation of $|V|^2$ distance metrics and their sorting [4]. We have tested the empirical runtime of the DBHT hierarchy computation for the PMFGs computed in Subsect. S.4.1 on MATLAB, and it scales as $\mathcal{O}(|V|^\alpha)$ with $\alpha \sim 1.7$.

S.4.4 Computation of shortest path lengths

We approached the shortest path problem (IV) by utilizing Johnson's algorithm in MATLAB BGL library, a graph library package for MATLAB [5]. Since PMFG is a sparse graph, Johnson's algorithm works in $\mathcal{O}(|V|^2 \log(|V|))$ [6].

S.4.5 Overall Runtime

We have computed empirically the overall time complexity of the DBHT technique on the simulated data sets described in Subsect. S.4.1, finding that it follows $\mathcal{O}(|V|^\alpha)$ with $\alpha \sim 2.7$, being dominated by the runtime to construct PMFG. Let us note that PMFG construction is presently not optimized and its optimization could lead to a consistent reduction of computational time.

References

1. Boyer JM and Myrvold WJ (2004) On the cutting edge: Simplified o(n) planarity by edge addition. *Journal of Graph Algorithms and Applications*, 8.

2. Song WM, Di Matteo T, and Aste T (2011) Nested hierarchies in planar graphs. *Discrete Applied Mathematics*, 159(17):2135 – 2146.

3. Wood DR (2007) On the maximum number of cliques in a graph. *Graph. Comb.*, 23:337–352.

Sample Cluster #	GO ID	corr p-value	Gene Count	GO description
1	22403	5.93E-20	25/58	cell cycle phase
:Gene Cluster 44	22402	3.78E-18	26/58	cell cycle process
	279	1.77E-16	21/58	M phase
	7049	8.78E-15	26/58	cell cycle
	51301	9.84E-11	16/58	cell division
	51726	1.12E-10	18/58	regulation of cell cycle
	278	1.19E-10	17/58	mitotic cell cycle
	6996	5.99E-10	27/58	organelle organization
	16043	4.30E-08	33/58	cellular component organization
	280	1.64E-07	12/58	nuclear division
	7067	1.64E-07	12/58	mitosis
	87	2.31E-07	12/58	M phase of mitotic cell cycle
	48285	2.54E-07	12/58	organelle fission
	6259	1.88E-06	15/58	DNA metabolic process
	6974	6.49E-06	13/58	response to DNA damage stimulus
	51321	1.11E-05	8/58	meiotic cell cycle
	75	1.50E-05	8/58	cell cycle checkpoint
	6281	3.75E-05	11/58	DNA repair
	44260	4.41E-05	34/58	cellular macromolecule metabolic process
	48522	9.05E-05	25/58	positive regulation of cellular process
	65009	1.48E-04	18/58	regulation of molecular function
	33554	1.69E-04	14/58	cellular response to stress
	51276	1.87E-04	13/58	chromosome organization
	79	2.05E-04	6/58	regulation of cyclin-dependent protein kinase activity
	7126	2.42E-04	7/58	meiosis
	51327	2.42E-04	7/58	M phase of meiotic cell cycle
	51716	2.92E-04	17/58	cellular response to stimulus
	50790	5.38E-04	16/58	regulation of catalytic activity
	48518	6.09E-04	25/58	positive regulation of biological process
	90304	7.63E-04	20/58	nucleic acid metabolic process
	6139	1.03E-03	22/58	nucleobase, nucleoside, nucleotide and nucleic acid metabolic process
	9987	1.13E-03	54/58	cellular process
	43170	1.50E-03	34/58	macromolecule metabolic process
	51340	1.54E-03	6/58	regulation of ligase activity
	7051	2.17E-03	5/58	spindle organization
	44237	2.65E-03	38/58	cellular metabolic process
	65003	3.28E-03	13/58	macromolecular complex assembly
	34641	3.42E-03	23/58	cellular nitrogen compound metabolic process
	51329	4.83E-03	6/58	interphase of mitotic cell cycle
	6310	5.41E-03	6/58	DNA recombination
	51325	6.05E-03	6/58	interphase
	43933	6.96E-03	13/58	macromolecular complex subunit organization
	6266	7.42E-03	3/58	DNA ligation
	42127	7.43E-03	14/58	regulation of cell proliferation
	48519	8.31E-03	22/58	negative regulation of biological process
	6807	8.92E-03	23/58	nitrogen compound metabolic process
4	50851	4.41E-02	3/33	antigen receptor-mediated signaling pathway
: Gene Cluster 109				
5	44260	3.16E-14	107/206	cellular macromolecule metabolic process
: Gene Cluster 1	43170	2.74E-11	110/206	macromolecule metabolic process
	44237	4.72E-10	123/206	cellular metabolic process
	43687	4.40E-09	52/206	post-translational protein modification
	44238	1.76E-08	124/206	primary metabolic process
	43412	1.11E-07	57/206	macromolecule modification
	6464	1.47E-07	55/206	protein modification process
	44267	3.12E-06	65/206	cellular protein metabolic process
	8152	4.17E-06	128/206	metabolic process
	50794	8.38E-06	131/206	regulation of cellular process
	6468	1.05E-05	31/206	protein amino acid phosphorylation
	90304	2.33E-05	49/206	nucleic acid metabolic process
	10468	2.74E-05	77/206	regulation of gene expression
	6796	4.94E-05	37/206	phosphate metabolic process
	6793	4.94E-05	37/206	phosphorus metabolic process
	16310	5.55E-05	33/206	phosphorylation
	34641	8.94E-05	60/206	cellular nitrogen compound metabolic process
	6139	1.23E-04	54/206	nucleobase, nucleoside, nucleotide and nucleic acid metabolic process
	31323	1.34E-04	89/206	regulation of cellular metabolic process
	51171	1.47E-04	77/206	regulation of nitrogen compound metabolic process
	10556	1.64E-04	74/206	regulation of macromolecule biosynthetic process
	16071	1.84E-04	21/206	mRNA metabolic process
	19219	2.31E-04	76/206	regulation of nucleobase, nucleoside, nucleotide and nucleic acid metabolic process
	45449	2.36E-04	69/206	regulation of transcription
	6807	2.73E-04	61/206	nitrogen compound metabolic process
	50789	3.65E-04	131/206	regulation of biological process
	60255	6.25E-04	81/206	regulation of macromolecule metabolic process
	7165	6.60E-04	54/206	signal transduction
	19538	1.09E-03	67/206	protein metabolic process
	31326	1.24E-03	74/206	regulation of cellular biosynthetic process
	80090	1.32E-03	83/206	regulation of primary metabolic process
	19222	1.35E-03	89/206	regulation of metabolic process
	9889	1.71E-03	74/206	regulation of biosynthetic process
	16070	2.21E-03	34/206	RNA metabolic process
	6357	6.65E-03	28/206	regulation of transcription from RNA polymerase II promoter
	7049	7.26E-03	29/206	cell cycle
7	48102	7.16E-03	2/30	autophagic cell death
:Gene Cluster 4				
9	6955	5.56E-09	21/75	immune response
: Gene Cluster 125	2376	7.82E-09	25/75	immune system process
	9611	2.20E-05	16/75	response to wounding
	6952	2.22E-05	17/75	defense response
	6950	4.28E-05	28/75	response to stress
	23052	5.59E-05	38/75	signaling
	50896	8.44E-05	41/75	response to stimulus
	6954	1.15E-04	12/75	inflammatory response
	6935	3.37E-04	9/75	chemotaxis
	42330	3.37E-04	9/75	taxis
	40011	5.96E-04	13/75	locomotion
	23033	1.55E-03	28/75	signaling pathway
	9607	4.73E-03	12/75	response to biotic stimulus
	22603	5.24E-03	10/75	regulation of anatomical structure morphogenesis
	7165	7.87E-03	25/75	signal transduction
	7166	9.01E-03	20/75	cell surface receptor linked signaling pathway
11				
(CLL Cluster)				
: Gene Cluster 102				

Table 2. Over-represented GO terms for each of the significant gene-clusters of sample-clusters 1, 5, 7, 9 (associated to DLBCL), 4 (associated to FL) and 11 (associated to CLL).

Clone name
*LyGDI=Rho GDP-dissociation inhibitor 2=RHO GDI 2; Clone=23
*LyGDI=Rho GDP-dissociation inhibitor 2=RHO GDI 2; Clone=1240974
*FLI-1=ERGB=ets family transcription factor; Clone=280882
*FLI-1=ERGB=ets family transcription factor; Clone=1354062
(Arp2/3 protein complex subunit p34-Arc (ARC34); Clone=1334980)
(Unknown UG Hs.28242 ESTs; Clone=1303641)
(Aconitase=mitochondrial protein; Clone=1353272)
(B-actin, 421-689; Clone=136)
(B-actin,177-439; Clone=137)
(Retinoblastoma-like 1 (p107); Clone=249725)
(B-actin, 657-993; Clone=145)
*actin=cytoskeletal gamma-actin; Clone=1240822
*Similar to nuclear protein NIP45=potentiates NFAT-driven interleukin-4 transcription; Clone=512953
actin=cytoskeletal gamma-actin; Clone=588637
*Adenine nucleotide translocator 2; Clone=291660
*Adenine nucleotide translocator 2; Clone=1241102
*Calmodulin 1 (phosphorylase kinase, delta); Clone=549080

Table 3. List of clones in gene-cluster 102, which corresponds to the most significant gene-cluster for sample-cluster 11 associated to CLL.

4. Day W (1996) Complexity theory: An introduction for practitioners of classification. In P. Arabie and L. Hubert, editors, *Clustering and Classification,*, pages 199–233. World Scientific Publishing Co. Inc.

5. Gleich D, Matlab bgl - graph library. Available: http://dgleich.github.com/matlab-bgl/. Accessed Jan 20 2012.

6. Johnson DB (1977) Efficient algorithms for shortest paths in sparse networks. *J. ACM*, 24:1–13.
